# A Natural-Like Synthetic Small Molecule Impairs Bcr-Abl Signaling Cascades and Induces Megakaryocyte Differentiation in Erythroleukemia Cells

**DOI:** 10.1371/journal.pone.0057650

**Published:** 2013-02-27

**Authors:** Silvia Turroni, Manlio Tolomeo, Gianfranco Mamone, Gianluca Picariello, Elisa Giacomini, Patrizia Brigidi, Marinella Roberti, Stefania Grimaudo, Rosaria Maria Pipitone, Antonietta Di Cristina, Maurizio Recanatini

**Affiliations:** 1 Department of Pharmacy and Biotechnology, University of Bologna, Bologna, Italy; 2 Interdepartmental Center of Research in Clinical Oncology and Department of Infectious Diseases, University of Palermo, Palermo, Italy; 3 Institute of Food Science, CNR, Avellino, Italy; European Institute of Oncology, Italy

## Abstract

Over the past years, we synthesized a series of new molecules that are hybrids of spirocyclic ketones as complexity-bearing cores with bi- and ter-phenyls as privileged fragments. Some of these newly-shaped small molecules showed antiproliferative, pro-apoptotic and differentiating activity in leukemia cell lines. In the present study, to investigate more in depth the mechanisms of action of these molecules, the protein expression profiles of K562 cells treated with or without the compounds **IND_S1, MEL_T1, IND_S7** and **MEL_S3** were analyzed using two-dimensional gel electrophoresis coupled with mass spectrometry. Proteome comparisons revealed several differentially expressed proteins, mainly related to cellular metabolism, chaperone activity, cytoskeletal organization and RNA biogenesis. The major results were validated by Western blot and qPCR. To attempt integrating findings into a cellular signaling context, proteomic data were explored using MetaCore. Network analysis highlighted relevant relationships between the identified proteins and additional potential effectors. Notably, qPCR validation of central hubs showed that the compound **MEL_S3** induced high mRNA levels of the transcriptional factors EGR1 and HNF4-alpha; the latter to our knowledge is reported here for the first time to be present in K562 cells. Consistently with the known EGR1 involvement in the regulation of differentiation along megakaryocyte lineage, **MEL_S3**-treated leukemia cells showed a marked expression of glycoprotein IIb/IIIa (CD41) and glycoprotein Ib (CD42), two important cell markers in megakaryocytic differentiation, together with morphological aspects of megakaryoblasts and megakaryocytes.

## Introduction

Chronic myeloid leukemia (CML) is a myeloproliferative disorder characterized by increased proliferation of the granulocytic cell line. The annual incidence is one or two cases per 100,000 adults with a slight male predominance. Up to 95% of CML patients harbour the t(9;22)(q34;q11) chromosomal translocation, cytogenetically visible as the Philadelphia (Ph) chromosome, which directs the expression of the constitutively active tyrosine kinase BCR-ABL. The chimeric BCR-ABL protein activates a variety of downstream effectors and signaling pathways, leading to growth factor-independent cell cycle progression, failure to differentiate, inhibition of apoptosis, alterations in cell-cell and cell-matrix interactions, and leukemogenesis [1].

The management of CML has been revolutionized in 2001 by the introduction of imatinib mesylate (Gleevec®), a potent tyrosine kinase inhibitor (TKI) rationally and specifically designed using the structure of the ATP-binding pocket of the ABL protein kinase [2]. Imatinib binds to and stabilizes the inactive form of BCR-ABL, blocking its autophosphorylation and downstream kinase activity. This induces hematologic, cytogenetic and molecular response in the majority of CML patients, through inhibition of proliferation and triggering of apoptosis of BCR-ABL-expressing cells. However, clinical resistance develops frequently, particularly in accelerated phase and blastic crisis of CML. This has led to the development of second-generation BCR-ABL-targeting molecules, that have been proved to be effective in nearly all imatinib-resistant BCR-ABL-positive leukemias [3], [4], [5]. Nevertheless, most of these new drugs do not work against leukemia cells bearing specific mutations [6], [7]. Moreover, TKIs are ineffective in patients who undergo blastic transformation, and unable to eradicate CML at the stem cell level, underscoring the need to pursue novel therapeutic strategies [6], [8]. In this regard, differentiation induction therapy has attracted universal attention as a promising approach to treat leukemia by turning abnormal cells back to differentiate and cease proliferation. The best proof of principle for such an approach has been the treatment of acute promyelocytic leukemia with all-*trans* retinoic acid [9]. Several attempts to emulate this success with other nuclear hormone ligands or different classes of substances, such as hematopoietic cytokines or compounds affecting the epigenetic landscape, have followed over the years but remained rather modest and disappointing [10]. Currently, research efforts are geared towards targeting signaling pathways that are chronically activated and critical for transformation of leukemia cells, for example by manipulating the transcription factors that govern the differentiation and lineage commitment of hematopoietic progenitors [8], [10], [11].

**Figure 1:**
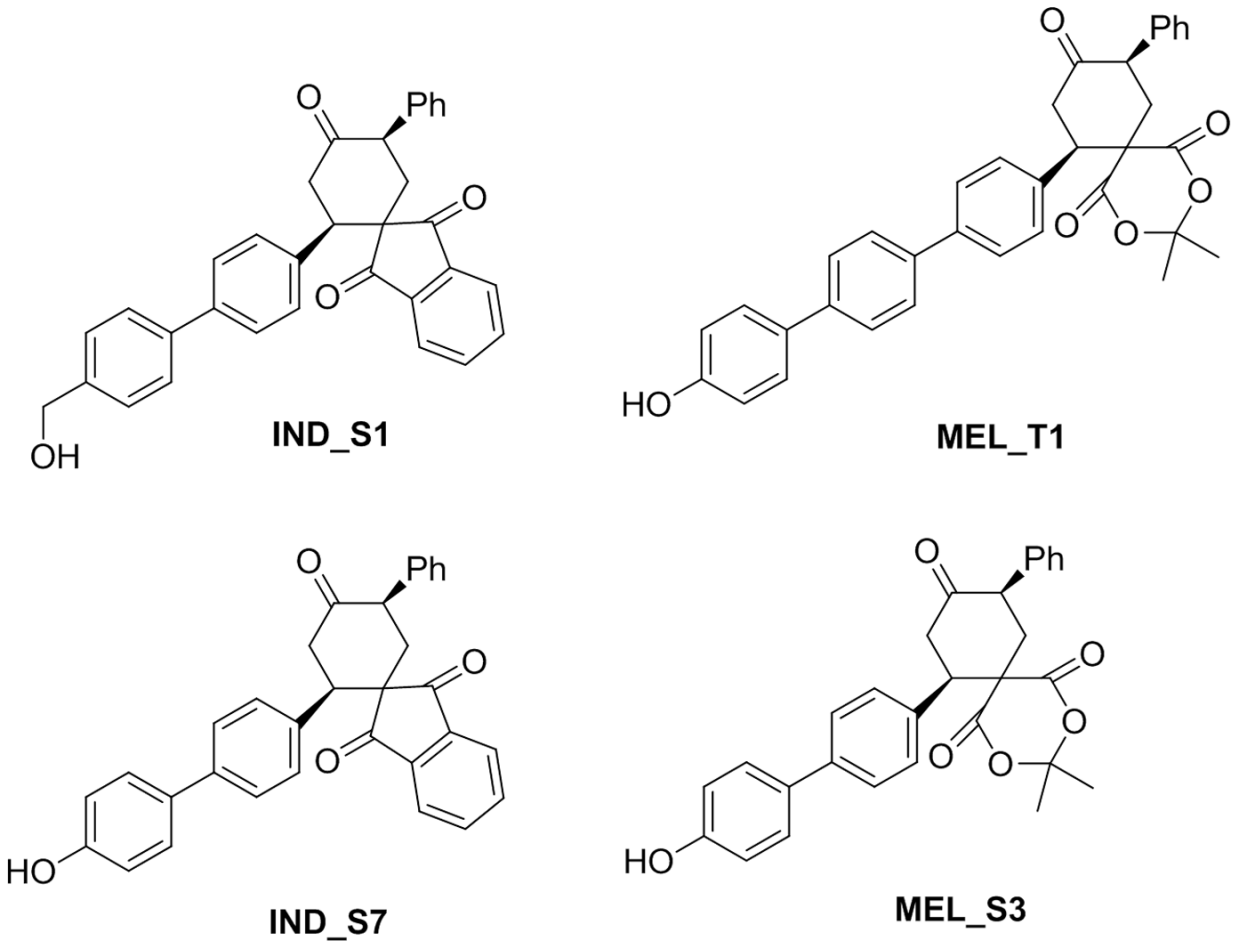
Synthetic natural-like biphenyl and terphenyl compounds.

In this context, over the past years through an integrated chemical biological strategy, we obtained four natural-like synthetic biphenyl and terphenyl compounds, **IND_S1**, **MEL_T1**, **IND_S7** and **MEL_S3** (Figure 1), able to interfere with the signaling cascades conferring apoptosis resistance and uncontrolled proliferation to BCR-ABL-expressing leukemia cells [12], [13]. To investigate more in depth the mechanisms of action of these molecules we believed that a proteomic approach could be a suitable strategy. Proteomics is an evolving technology platform that is gaining widespread use in drug discovery 14,15. Common applications include target identification and validation, identification of molecular biomarkers and investigation into mechanisms of drug action or toxicity. In the present study, a comparative proteomic approach based on two-dimensional gel electrophoresis (2-DE) and MALDI-TOF mass spectrometry was carried out to define the protein expression profiles of **IND_S1**-, **MEL_T1**-, **IND_S7**- and **MEL_S3**-treated and -untreated K562 cells. The major results were validated by Western blot and quantitative PCR (qPCR) analysis. Differentially expressed proteins were further investigated using MetaCore pathway analysis program to attempt integrating the findings into a cellular signaling context. The central hubs of significant subnetworks were verified by qPCR. With this strategy we identified several transcription factors dysregulated at the mRNA level in K562 cells treated with the four compounds. Since most of them are known to be essential for hematopoiesis, the differentiation potential of the molecules was explored by immunofluorescence flow cytometry. Noteworthy, the molecule **MEL_S3** was able to induce megakaryocytic differentiation in K562 cells at a rate correlated to its ability to modulate the expression of EGR1 gene, which is known to be involved in this kind of differentiation [16].

## Results

### Proteome Profiling of K562 Cells Treated with Four Synthetic Small Molecules

**Figure 2:**
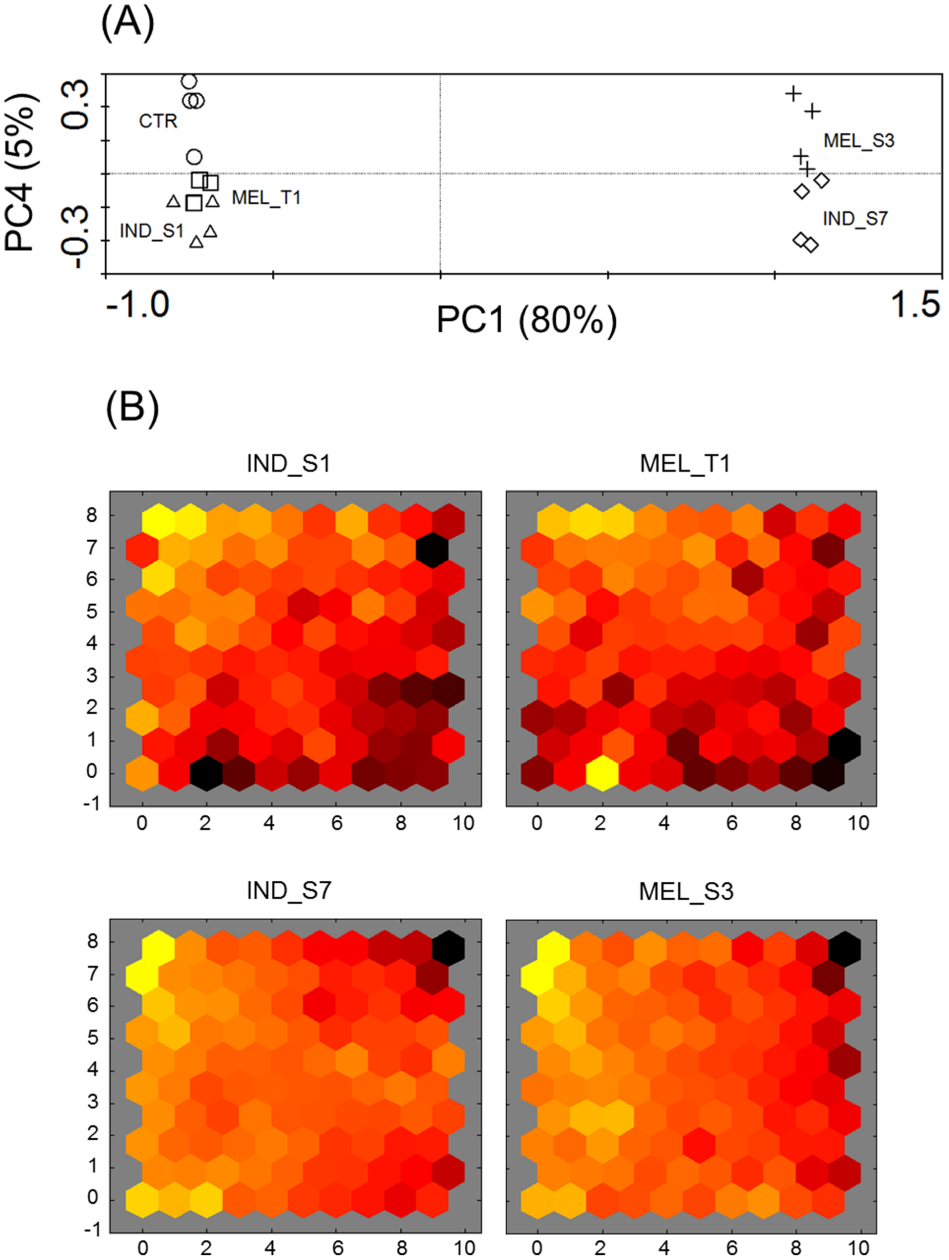
Multivariate analysis of proteomic data. (A) PCA plot of the expression profiles from K562 cells untreated (circles) and after 24-h exposure to **IND_S1** (triangles), **MEL_T1** (squares), **IND_S7** (diamonds), **MEL_S3** (crosses). Log-transformed data were used. Each symbol represents a 2-DE gel from each treatment group. First and fourth ordination axes are plotted explaining 80 and 5% of the overall variance in the dataset, respectively. (B) Visualization of the SOM component planes of proteome data for all treatment series. Ratios between expression levels in K562 cells treated with the four synthetic small molecules and control cells were calculated and log_2_-transformed. Each presentation illustrates the weights that connect each input to each of the artificial neurons, resulting in a sample-specific proteome-wide map (darker colors represent larger weights). All figures are linked by position: in each display, the hexagon in a certain position corresponds to the same map unit.

To identify the proteins whose expression was responsive to the synthetic small molecules **IND_S1, MEL_T1, IND_S7** and **MEL_S3**, protein extracts from K562 cells grown for 24 h in the presence or absence of 30 µM of each compound were subjected to a 2-DE-based analysis. Representative silver-stained 2-DE gels of control and treated K562 cells are shown in Figure S1. On average, around 900 spots were detected on each analytical gel and analyzed by PDQuest software. PCA was applied to the entire 2-DE dataset to disclose differences in the protein patterns of K562 cells exposed to the four synthetic small molecules. As shown in the PCA plane of Figure 2A, the four compounds originated four fairly distinct groupings. PCA accounted for about 85% of the total variance (80 and 5% for PC1 and PC4 axes, respectively). Replicate 2-DE gels were closely grouped, indicating similarity in the spot maps. In particular, samples from K562 cells treated with **MEL_T1** were found closer together, suggesting less inter-gel variation respect to the other samples. K562 cells incubated with **IND_S1** and **MEL_T1** showed no clear separation with each other whereas, according to PC1, the largest difference existed between **IND_S7**- and **MEL_S3**-treated K562 cells and all the other samples. Analogously, the unsupervised SOM cluster analysis offered a global view of the protein expression profiles. SOM is a specific architecture of artificial neural networks, consisting of a low-dimensional interconnected grid of neurons, which allow to partition input data into related subsets. As shown by the SOM component planes of Figure 2B, the treatment with **IND_S1** and **MEL_T1** resulted in quite distinct connection patterns whereas the SOM outputs from **IND_S7**- and **MEL_S3**-treated K562 cells resembled each other, suggesting similar trends in protein expression profiles. Interestingly, the main differences among the four compounds were visualized in the bottom left and right corners of the connection patterns, which may account for molecule-specific mechanisms of protein regulation.

**Figure 3:**
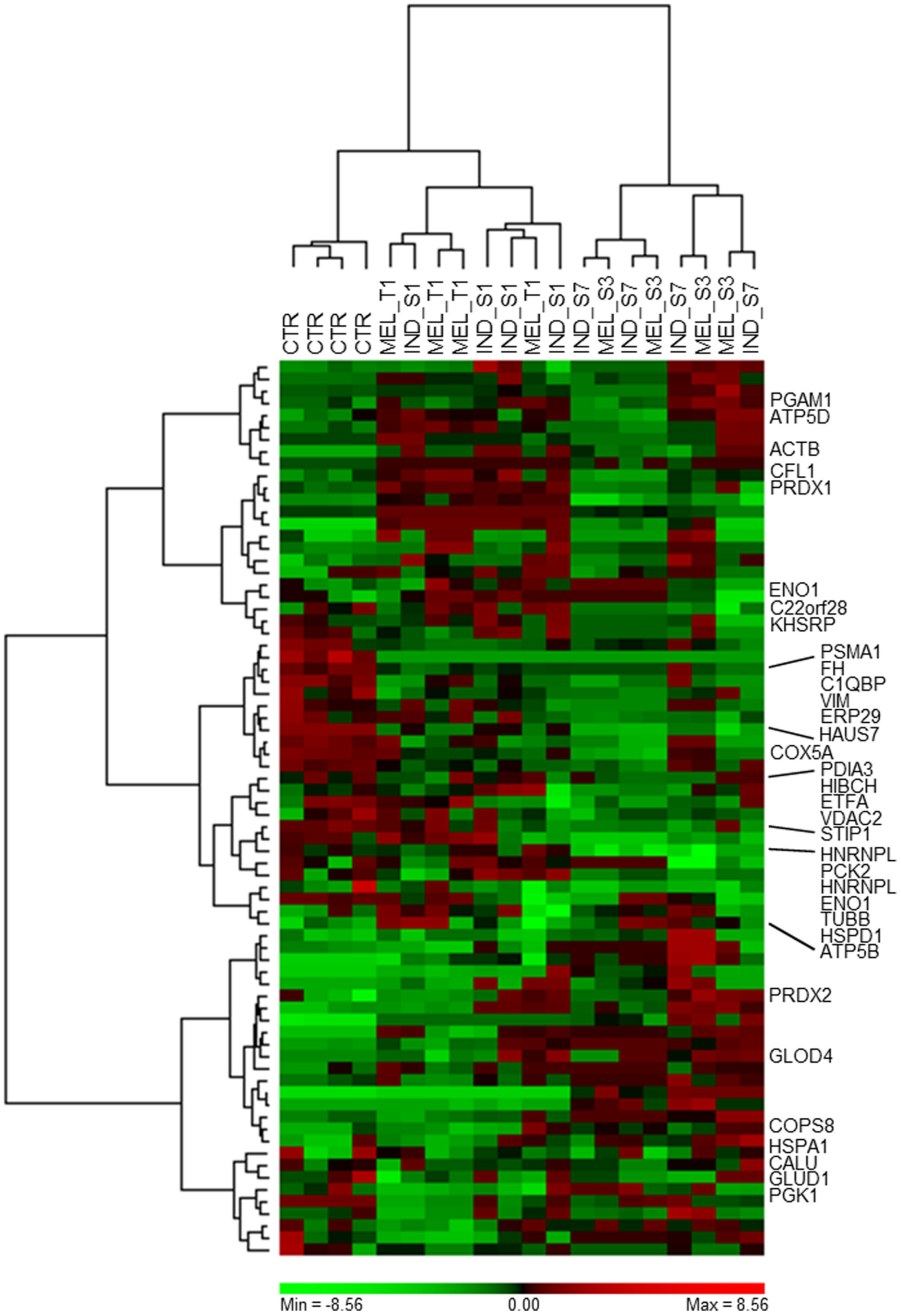
Two-way hierarchical clustering of the 74 differentially expressed protein spots between K562 cells treated with the four synthetic small molecules and control cells. Pearson’s dissimilarity as distance measure and Ward’s method for linkage analysis were used. Log_2_ ratios are color coded as indicated. Names of the identified protein spots are shown on the right (see Table 1).

Multivariate analysis was complemented with the analysis of individual protein spots using Student’s t-test and the non-parametric Mann-Whitney test. Following these combined approaches, 74 spots were found to be significantly altered in their protein abundance by at least two-fold between control and treated K562 cells (*P*<0.05). The largest number of differentially expressed spots was achieved for K562 cells exposed to **IND_S7** and **MEL_S3**, with approximately the same number of up- and down-regulated protein spots. Out of these differential spots, 49 were in common and exhibited the same trend of expression. Interestingly, 27/74 (36.5%) spots increased (16 spots) or decreased (11 spots) after treatment in all the 2-DE gels included in the analysis set, suggesting the existence of non-specific mechanisms of action, common to the four synthetic small molecules. Conversely, contrasting variations in protein abundance among treated K562 cells were found for 19 spots, probably representing compound-specific proteomic signatures, which may account for different biological effects. Differential spots were clustered employing Ward’s minimum variance method over a Pearson distance-based dissimilarity matrix (Figure 3). Replicate gels were mostly grouped in separate subclusters or separated by a short distance. As PCA and SOM analysis showed before, a clear difference was observed between **IND_S7**- and **MEL_S3**-treated K562 cells and all the other samples, suggesting that the major changes in protein expression took place after exposure to these compounds. Looking at the row clustering of the heat map, different expression dynamics of protein spots among the samples could be distinguished. In particular, three main trends of expression were identified, the first related to proteins whose levels were up-regulated in K562 cells incubated with **IND_S1** and **MEL_T1**, the second including protein spots that tended to decline in **IND_S7**- and **MEL_S3**-treated K562 cells and the last describing those increasing after exposure to **IND_S7** and **MEL_S3**.

**Figure 4:**
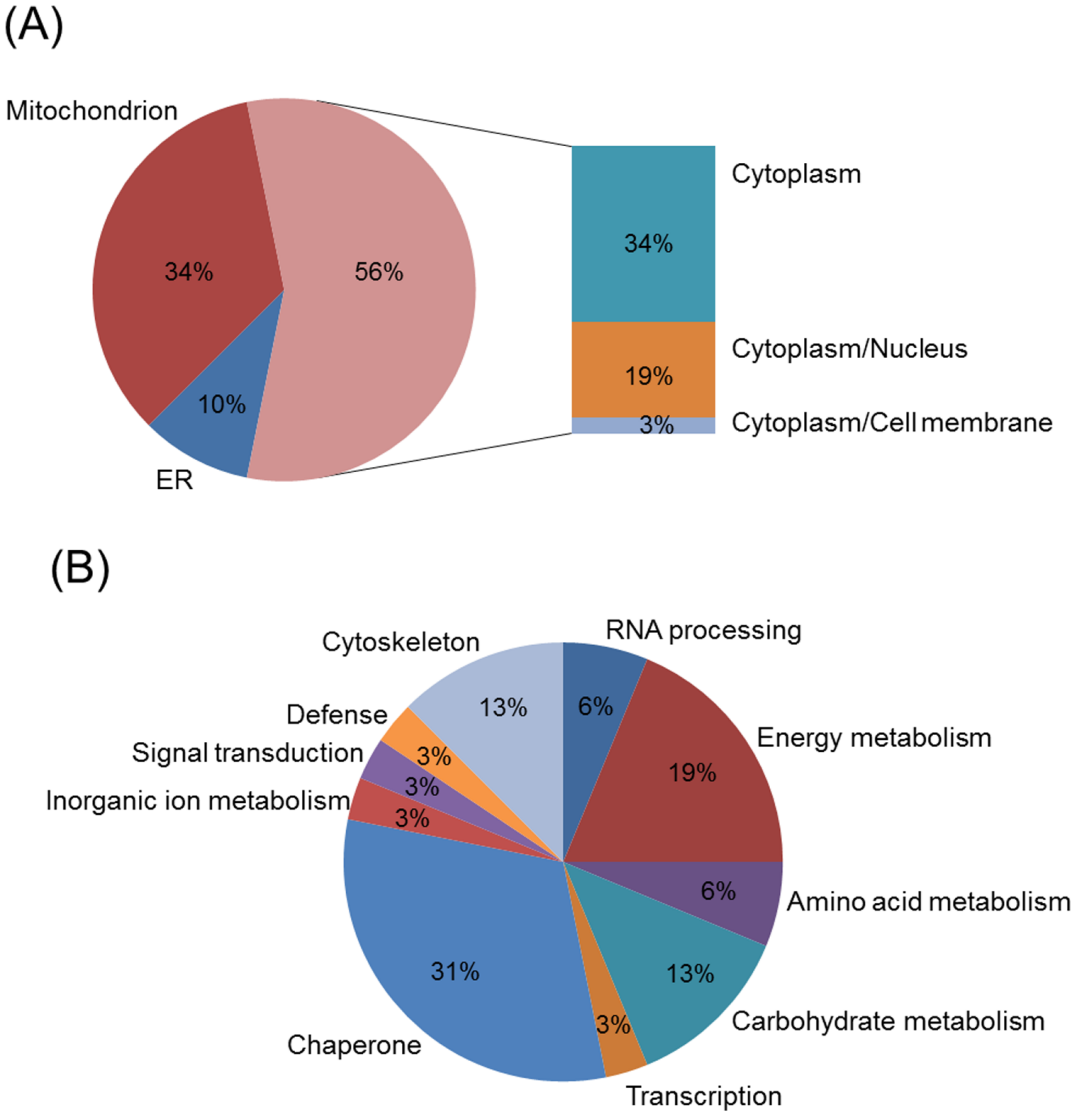
Pie chart distribution of the identified proteins according to cellular location (A) and biological function (B). Proteins were categorized using information from the Swiss-Prot, GO and COG databases. When proteins were associated with more than one function, one category was chosen arbitrarily. ER, endoplasmic reticulum.

Interesting spots were excised from preparative gels for protein identification by MALDI-TOF MS analysis. Following a Mascot database search using the acquired MS data, 34/74 (46%) protein spots were identified, corresponding to 32 unique proteins (Table 1). Each identified protein was assigned to a cellular localization based on information from the Swiss-Prot and GO databases. As shown in Figure 4A, the majority were cytoplasmic and mitochondrion proteins. With the exception of enolase, which is known to be localized also at the cell membrane level, no membrane proteins were identified, possibly as a consequence of their general poor solubilization. The identified proteins were further grouped into different classes based on biological function using COG database (Figure 4B). Most of significantly modulated proteins were related to cellular metabolism (41%). Chaperone activity accounted for 31% whereas 12% were categorized as having a major role in transcriptional regulation and signal transduction. Cytoskeletal proteins occupied 13% of the identified protein set.

**Table 1:** List of differentially expressed protein spots identified by MALDI-TOF MS.

Spot ID	Gene name	Protein name	Swiss-Prot Acc. No.	Theoretical		Sequence coverage (%)	MASCOT score	Matching peptides	Fold change		COG	Subcellular location
				MW	pI				IND_S1/MEL_T1	IND_S7/MEL_S3		
1	KHSRP	Far upstream element-binding protein 2	Q92945	73.12	6.85	50	268	42	NS	−24.0/−15.5	K	Nucleus Cytoplasm
2	HSPA1	Heat shock 70 kDa protein1A/1B	P08107	70.05	5.48	50	95	24	−2.1/−4.2	−2.1/−2.4	O	Cytoplasm
3	PCK2	Phosphoenolpyruvate carboxykinase [GTP], mitochondrial	Q16822	70.73	7.57	39	169	21	NS	−26.8/−11.2	C	Mitochondrion
4	HNRNPL	Heterogeneous nuclear ribonucleoprotein L	P14866	64.13	8.46	32	107	18	NS	−5.8/−2.5	A	Nucleus Cytoplasm
5	HNRNPL	Heterogeneous nuclear ribonucleoprotein L	P14866	64.13	8.46	39	129	23	NS	−14.3/−23.8	A	Nucleus Cytoplasm
6	HSPD1	Mitochondrial heat shock60kDa protein 1 variant 1	B3GQS7	60.68	5.83	38	105	9	NS/2.4	NS	O	Cytoplasm
7	PDIA3	Protein disulfide-isomerase A3	P30101	56.78	5.98	55	233	31	−2.2/NS	−4.4/NS	O	ER
8	STIP1	Stress-induced-phosphoprotein1	P31948	62.64	6.40	33	129	15	−4.6/NS	−2.3/−2.4	O	Nucleus Cytoplasm
9	C22orf28	tRNA-splicing ligase RtcB homolog	Q9Y3I0	55.21	6.77	47	161	20	NS	−6.3/−5.3	A	Cytoplasm
10	GLUD1	Glutamate dehydrogenase 1, mitochondrial	P00367	61.40	7.66	33	154	13	NS	2.6/−22.7	E	Mitochondrion
11	TUBB	Tubulin beta chain	P07437	49.67	4.78	70	199	30	−2.6/NS	−4.0/−4.6	Z	Cytoplasm
12	ATP5B	ATP synthase subunit beta, mitochondrial	P06576	56.56	5.26	41	197	13	4.4/5.4	4.4/3.3	C	Mitochondrion
13	CALU	Calumenin	O43852	37.11	4.47	50	89	9	NS	2.1/−26.6	TU	ER
14	VIM	Vimentin	P08670	53.65	5.06	65	218		NS	−9.5/−3.6	Z	Cytoplasm
15	HAUS7	HAUS augmin-like complex subunit 7	Q99871	40.78	4.73	39	130	12	NS	−4.6/−4.8	O	Cytoplasm
16	ENO1	Alpha-enolase	P06733	47.17	7.01	30	102	11	NS	−18.5/NS	G	Cytoplasm Cell membrane
17	ENO1	Alpha-enolase	P06733	47.17	7.01	32	127	14	−8.2/−5.4	−27.6/−24.7	G	Cytoplasm Cell membrane
18	FH	Fumarate hydratase, mitochondrial	P07954	54.64	8.85	48	139	22	−3.6/−2.4	−10.0/−9.4	C	Mitochondrion
19	PGK1	Phosphoglycerate kinase 1	P00558	44.61	8.30	39	146	13	−3.2/−5.6	−7.2/−2.6	G	Cytoplasm
20	HIBCH	3-hydroxyisobutyryl-CoA hydrolase, mitochondrial	Q6NVY1	43.48	8.38	37	100	10	2.2/2.6	NS	E	Mitochondrion
21	GLOD4	Glyoxalase domain-containing protein 4	Q9HC38	34.79	5.4	27	86	7	16.9/6.3	8.7/12.1	G	Mitochondrion
22	ETFA	Electron transfer flavoprotein subunit alpha, mitochondrial	P13804	35.08	8.62	49	10	12	−4.0/NS	−3.0/−4.4	C	Mitochondrion
23	VDAC2	Voltage-dependent anion-selective channel protein 2	P45880	31.57	7.50	44	99	8	−2.0/NS	−6.3/−2.7	P	Mitochondrion
24	C1QBP	Complement component 1 Q subcomponent-binding protein, mitochondrial	Q07021	31.36	4.74	42	83	11	−3.6/−3.7	−5.2/−3.3	V	Mitochondrion
25	PSMA1	Proteasome subunit alpha type-1	P25786	29.56	6.15	32	98	8	−6.6/−2.5	−10.0/−10.0	O	Nucleus Cytoplasm
26	ACTB	Actin, cytoplasmic 1	P60709	41.74	5.29	33	115	7	28.6/NS	20.1/22.2	Z	Cytoplasm
27	ERP29	Endoplasmic reticulum resident protein 29	P30040	28.99	6.77	39	93	16	NS	−3.7/−5.0	O	ER
28	PGAM1	Phosphoglycerate mutase 1	P18669	28.80	6.67	53	123	11	2.6/2.4	2.4/3.3	G	Cytoplasm
29	PRDX2	Peroxiredoxin-2	P32119	21.89	5.66	40	107	9	2.7/NS	2.9/3.8	O	Cytoplasm
30	COPS8	COP9 signalosome complex subunit 8	Q99627	23.23	5.25	38	114	7	8.4/17.1	10.3/12.0	OT	Nucleus Cytoplasm
31	PRDX1	Peroxiredoxin-1	Q06830	22.11	8.27	47	173	10	3.3/3.6	NS	O	Cytoplasm
32	CFL1	Cofilin-1	P23528	18.50	8.22	66	114	9	3.7/3.2	NS	DZ	Nucleus Cytoplasm
33	ATP5D	ATP synthase subunit delta, mitochondrial	P30049	17.49	5.38	43	108	8	2.2/2.2	2.1/2.1	C	Mitochondrion
34	COX5A	Cytochrome c oxidase subunit 5A, mitochondrial	P20674	16.76	6.30	51	89	6	−2.3/−2.2	−4.0/−4.3	C	Mitochondrion

NS, not statistically significant. ER, endoplasmic reticulum. A, RNA processing and modification; C, Energy production and conversion; D, Cell cycle control, cell division, chromosome partitioning; E, Amino acid transport and metabolism; G, Carbohydrate transport and metabolism; K, Transcription; O, Post-translational modification, protein turnover, chaperones; P, Inorganic ion transport and metabolism; T, Signal transduction mechanisms; U, Intracellular trafficking, secretion, and vesicular transport; V, Defense mechanisms; Z, Cytoskeleton.

### Confirmation of Selected Differentially Expressed Proteins

**Figure 5:**
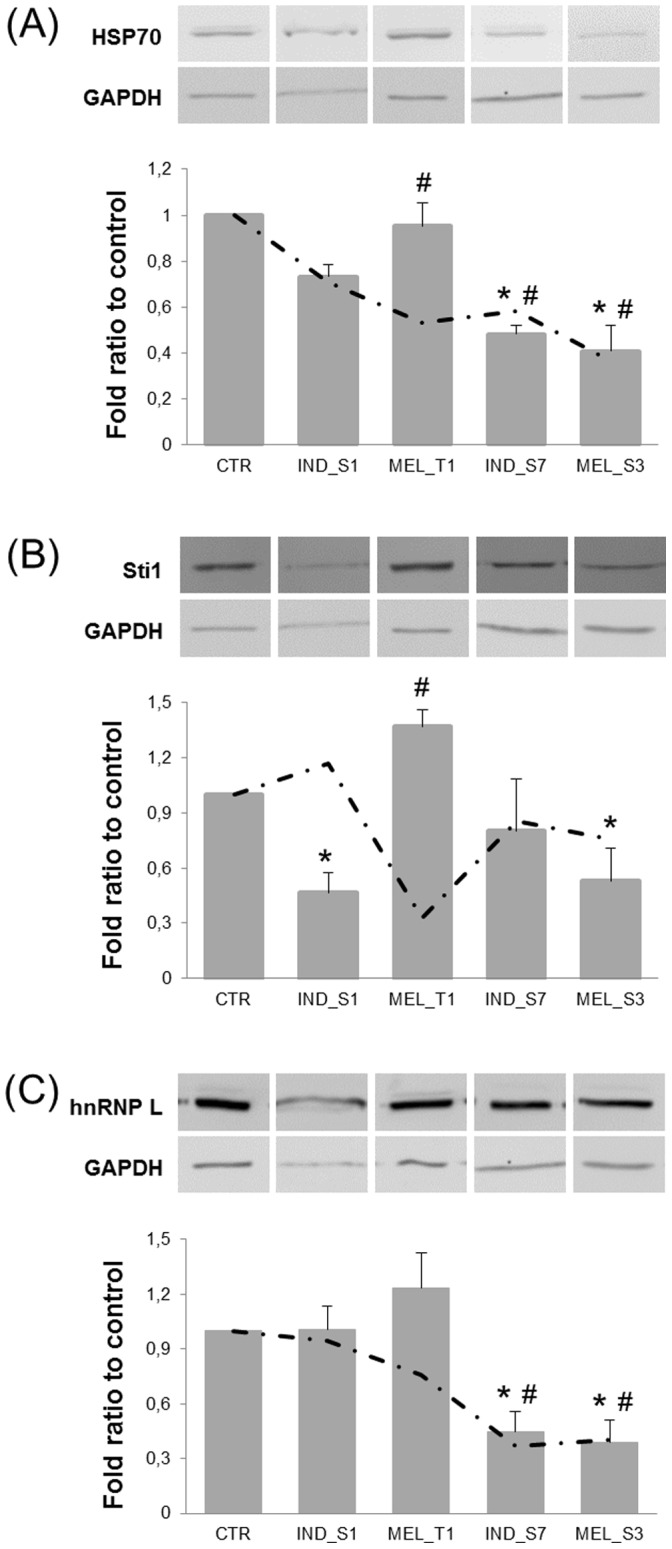
Western blot validation and qPCR determination of mRNA levels of HSP70 (A), Sti1 (B) and hnRNP L (C). Top, Representative immunoblots of each protein and GAPDH, as a loading control. Bottom, Bar graphs showing the densitometric analysis of Western blots. For each treatment series, protein values were normalized to GAPDH and expressed as a ratio relative to control K562 cells. Data are presented as mean ± SD from at least 3 separate experiments. *, *P*<0.05 vs control. Dash-dotted line represents transcription levels of genes encoding HSP70, Sti1 and hnRNP L, as determined by qPCR. The transcript amounts were normalized against 3 housekeeping genes (TBP, GAPDH and BCR) following the normalization strategy proposed by Vandesompele *et al.* [17]. Data are presented as mean ± SD of the fold ratio between treated and control K562 cells, derived from at least 2 separate experiments. ^#^, *P*<0.05 vs control.

To independently evaluate the reliability of the proteomic results, semi-quantitative Western blots were performed for three proteins that exhibited moderate abundance changes in the 2-DE maps. As shown in Figure 5, expression changes of HSP70 (spot no. 2) and Sti1 (spot no. 8) were generally consistent with 2-DE results. For hnRNP L (spots no. 4, 5), even if the direction of the observed variations using both methods was the same, the magnitude of the change was substantially different. In particular, analysis of densitometric values of the Western blot bands associated to hnRNP L from K562 cells exposed to **IND_S7** and **MEL_S3** revealed a down-regulation ranging from two- to three-fold, in sharp contrast with the >14-fold decrease found for spot no. 5. Considering that hnRNP L was detected on 2-DE gels as two distinct spots along the horizontal axis, which may reflect post-translational modification-induced charge alterations, such discrepancies could suggest the modulation of a specific hnRNP L variant rather than the total protein level in **IND_S7**- and **MEL_S3**-treated K562 cells.

To determine whether these proteins were also dysregulated at the transcriptional level, their mRNA transcripts were quantified by qPCR. PCR efficiency was close to 2 (within 75–107%) for all housekeeping and target gene primers (Table S1). Correlation coefficients were >0.99. Assay reproducibility and reliability were evaluated by repeating cDNA synthesis and qPCR two times under identical conditions. The intra-assay CVs were about 5% and 2% for cDNA synthesis and qPCR, respectively. The inter-replicate CVs were lower than 10%. Reference gene stability was assessed using geNorm and NormFinder. Both algorithms identified TBP, GAPDH and BCR as the most stably expressed control genes, whereas ABL was found to be the worst-scoring one (Table S2). Since the pairwise variation between two sequential NFs containing an increasing number of genes was below the cutoff value of 0.15 [17], the three best-performing housekeeping genes were used as internal controls for geometric averaging. The relative abundances of target gene transcripts were thus determined following the normalization strategy outlined by Vandesompele *et al.* (2002). For each selected protein the fold ratio of mRNA expression in treated K562 cells compared to control samples, which were arbitrarily set to 1, was estimated. For HSP70, the mRNA regulation profiles were generally coinciding with the protein patterns. In particular, transcript levels were significantly reduced after 24-h treatment with all compounds except for **IND_S1** (Figure 5A). Interestingly, such reduction was measured already 6 h after exposure (data not shown). Conversely, the abundance of the transcript encoding Sti1 was constant throughout the entire dataset but reached a three-fold lower level after 24-h exposure to **MEL_T1** compared to control (Figure 5B). hnRNP L mRNA expression was consistent with proteomic data, with a significant decrease only in **IND_S7**- and **MEL_S3**-treated K562 cells (Figure 5C). Differently from HSP70, the transcriptional levels of hnRNP L gradually decreased over time (data not shown). Since a major role of hnRNP L in modulating the alternative splicing of caspase-9 has been recently demonstrated [18], caspase-9 mRNA expression was also measured. As shown in Figure S2, caspase-9 transcript levels were highly dysregulated, with a >8-fold increase after **IND_S1** and **MEL_S3** exposure.

To further verify proteomic results at the mRNA level, other five proteins were arbitrarily selected and subjected to qPCR (Figure S2). As expected, different correlation patterns between mRNA and protein abundance were identified. Similar trends of variation were observed for HCOP9 (spot no. 30), whose transcript levels significantly increased after 24-h treatment with all four synthetic small molecules. On the contrary, despite the wide variations at the protein level, HSPC117 (spot no. 9) and GLOD4 (spot no. 21) were found to be transcriptionally unaltered, suggesting that post-translational events contributed to the protein changes. An inverse correlation, with up-regulation of mRNA concurrent with down-regulation of protein expression was observed for vimentin (spot no. 14), probably reflecting degradation events. Inconsistent results between 2-DE and qPCR analysis were obtained for HAUS7 (spot no. 15).

### Network Analysis of Identified Proteins and Evaluation of Critical Protein Changes Using qPCR

**Figure 6:**
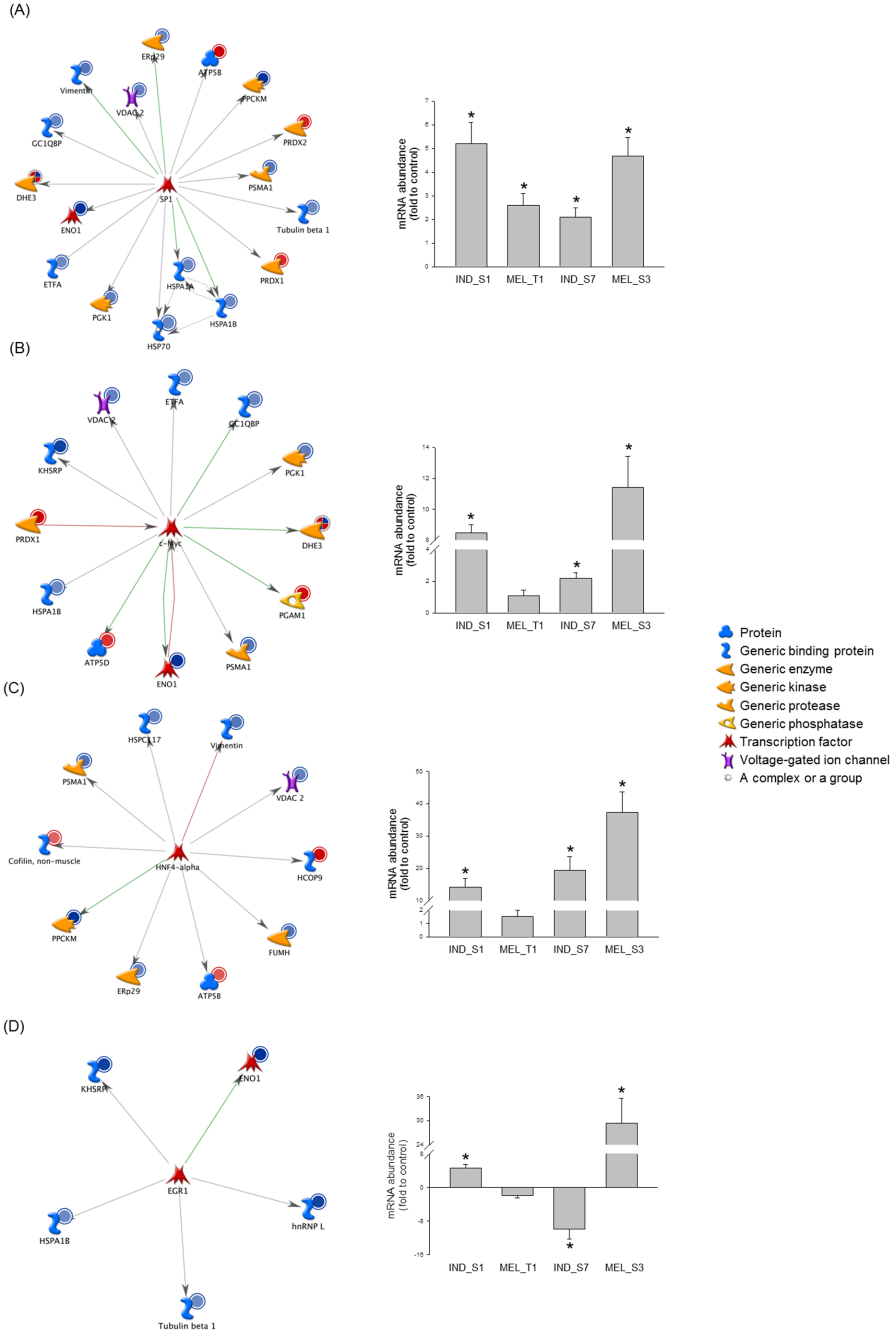
Network analysis of differentially expressed proteins and validation of central hubs at the mRNA level. The transcription regulation networks initiated through activation of SP1 (A), c-Myc (B), HNF4-alpha (C) and EGR1 (D) are shown together with the mRNA abundances of these transcription factors in K562 cells treated with the four synthetic small molecules. Network proteins are visualized by proper symbols, which specify the functional nature of the protein (network caption). Red, green and gray arrows indicate negative, positive and unspecified effects, respectively. Red and blue circles indicate up- and down-regulated proteins in the treatment series, respectively. Relative transcription levels were quantified by qPCR according to Vandesompele *et al.* [17] (see text for details). The bar graph plots mean ± SD of the fold ratio between treated and control K562 cells, derived from at least 2 separate experiments. *, *P*<0.05.

Pathways and networks that involved differentially expressed proteins from 2-DE gels were analyzed using MetaCore. Pathway enrichment analysis revealed that the majority of identified pathway maps were related to glycolysis and gluconeogenesis, as well as cytoskeleton remodeling (Figure S3A). Additionally, enrichment in the ontology of functional processes, as defined by “GeneGo process networks”, was calculated. As it would be expected, the top-scoring process was “regulation of cytoskeleton rearrangement” (Figure S3B). To map interaction between proteins, the shortest direct paths were analyzed using the “analyze network” algorithm. Based on the functional subnetworks built, the differentially expressed proteins after K562 cell treatment with the four synthetic small molecules were primarily involved in regulation of apoptosis (*P* = 4.16×10^−21^), as well as regulation of cellular biosynthetic process and RNA metabolic process (*P* = 8.49×10^−11^) (Table S3). Four protein objects [calumenin (spot no. 13), GLOD4, HIBCH (spot no. 20) and HSPC117] were not connected to any of the network hubs. To determine specific transcription factors that could drive the proteome changes in K562 cells exposed to the different compounds, a variant of the “shortest paths” algorithm was used to generate transcriptional regulation networks (Table S4). SP1 was ranked #1, with 15 targets among the 32 identified proteins from 2-DE gels (Figure 6A). Other top-scoring transcriptional regulators were c-Myc (Figure 6B) and HNF4-alpha (Figure 6C). Interestingly, the regulation mechanisms of almost all the HNF4-alpha targets were unknown. EGR1, a crucial gene in megakaryocyte differentiation of K562 cell line [19], [20], [21], was shown to transcriptionally regulate 5 differentially expressed proteins, HSP70, hnRNP L, ENO1 (spots no. 16, 17), tubulin beta (spot no. 11) and KHSRP (spot no. 1) (Figure 6D).

To validate the transcription regulation networks, the mRNA levels of the most interesting central hubs were measured by qPCR. Results confirmed a significant dysregulation for SP1, c-Myc, HNF4-alpha, EGR1 (Figure 6), HSF1, androgen receptor, C/EBPbeta and NF-YB (Figure S4), though different patterns of mRNA abundance were identified. In particular, transcripts encoding SP1 and C/EBPbeta increased after 24-h treatment with all compounds, even if C/EBPbeta increase was measurable already 6 h after exposure and reached a peak value after 15 h (fold ratio, **IND_S1**, 8.6; **MEL_T1**, 2.5; **IND_S7**, 8.0; **MEL_S3**, 6.8). Androgen receptor and NF-YB were moderately but significantly modulated in response to a single compound, whilst no significant changes in the transcriptional levels were detected for the other NF-Y subunits A and C (data not shown). Significantly higher levels compared to control K562 cells were measured for c-Myc and HNF4-alpha in **IND_S1**-, **IND_S7**- and **MEL_S3**-treated cells. Interestingly, both transcripts gradually increased over time, reaching the highest levels in K562 cells exposed to **MEL_S3** (fold ratio, c-Myc, 11.4; HNF4-alpha, 37.2). **MEL_S3**-treated K562 cells also showed a dramatic increase of mRNA amount of both EGR1 (fold ratio 29.3) and HSF1 (fold ratio 23.1). Notably, different change directions were found for EGR1 in response to the four small synthetic molecules.

### Differentiation Marker Analysis of K562 Cells Treated with the Four Synthetic Small Molecules

**Figure 7:**
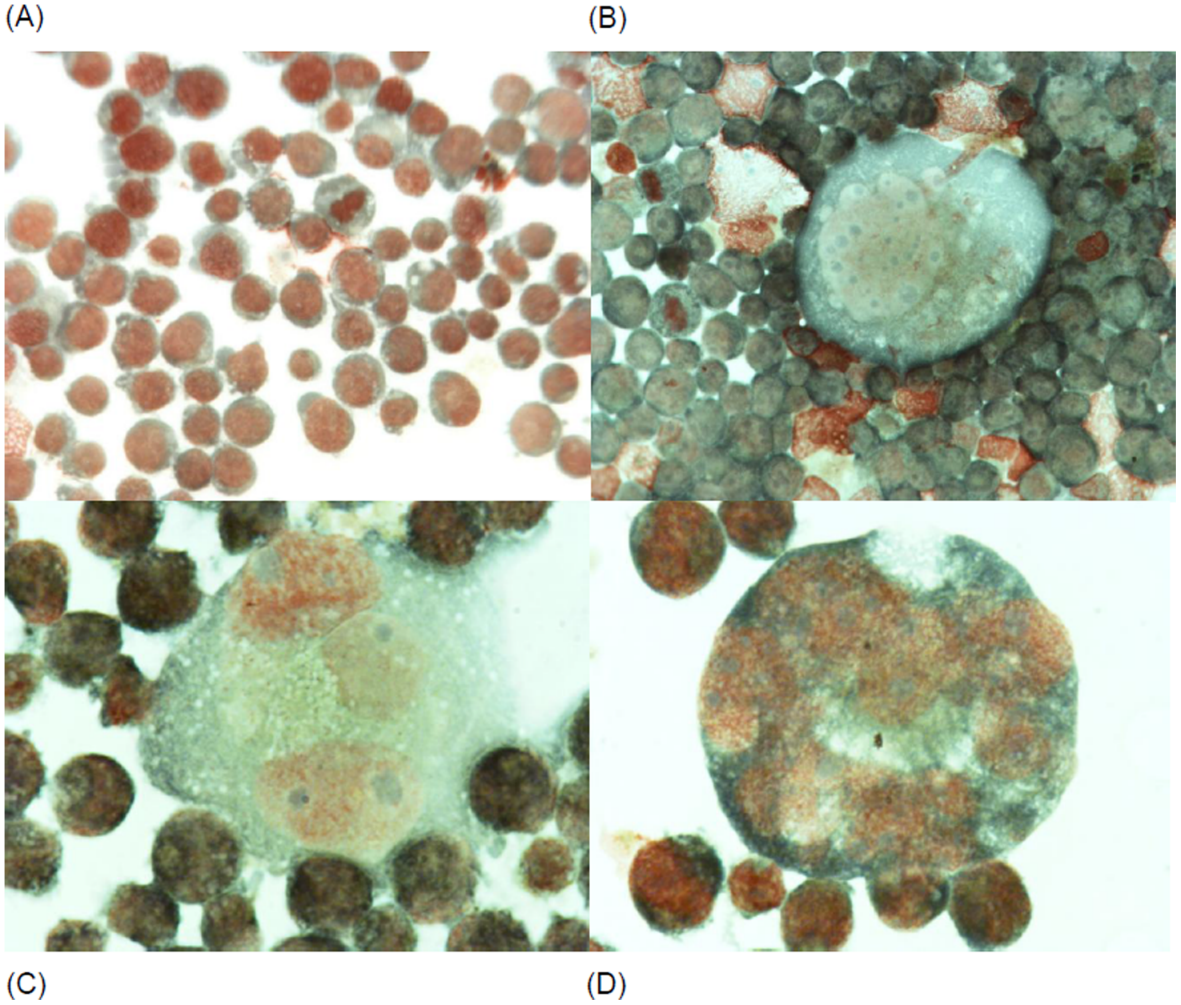
Morphological changes observed in K562 cells after 72-h exposure to 15 µM of MEL_S3. (A) Control; (B, C and D) **MEL_S3-**treated K562 cells with the presence of large cells with megakaryocytic morphological aspects.

Since K562 cells are capable of multilineage differentiation [16] and EGR1 is implicated in megakaryocyte differentiation [21], [22] we investigated the ability of **IND_S1**, **MEL_T1**, **IND_S7** and **MEL_S3** to induce differentiation of K562 cells along the megakaryocyte lineage; erythroid differentiation was also investigated. Cells were exposed for 72 h to 15 µM of each compound. Megakaryocyte differentiation was determined evaluating the expression of glycoprotein IIb/IIIa (CD41), which is normally present on platelets and early promegakaryoblasts. Erythroid differentiation was estimated by evaluating the cell surface expression of glycophorin A (CD235a), a major transmembrane sialoglycoprotein expressed on red blood cells and erythroid precursors. As shown in Figure S5A (panel a), **MEL_S3** induced a marked megakaryocyte differentiation based on induction of glycoprotein IIb/IIIa. Of interest, the expression of CD235a was slightly decreased in K562 cells treated with **MEL_S3** (Figure S5B, panel a). Also **IND_S1** was able to differentiate K562 cells in megakaryocytes (Figure S5A, panel c) but the expression of glycoprotein IIb/IIIa was lower than that observed in **MEL_S3**-treated K562 cells. In contrast, **MEL_T1** and **IND_S7** did not induce any modulation of either glycoprotein IIb/IIIa or glycophorin A on the cell surface membrane. These data are consistent with the ability of **MEL_S3** and **IND_S1** to increase the expression of the EGR1 gene. Megakaryocyte differentiation in K562 cells treated with **MEL_S3** was also investigated by morphological examination and expression evaluation of glycoprotein Ib (GPIb), also known as CD42. As shown in Figure 7, microscope examination of **MEL_S3**-treated K562 cells showed many large cells with morphological characteristics of megakaryocyte. Moreover, treated cells showed an increased expression of CD42 (Figure S6 panel b). Finally, the differentiation effects of **MEL_S3** were tested in HEL cells that, like to K562, are endowed with erythroid and megakaryocytic properties. As shown in Figure S6 (panel c, d), an increased expression of both CD41 and CD42 was observed after exposure to **MEL_S3** in HEL cells.

## Discussion

Chronic myeloid leukemia (CML) is a disease driven by a single molecular defect, the BCR-ABL translocation, which results in the activation and dysregulation of a large number of signaling pathways, speeding up cell division and incorporation of genetic abnormalities [23]. The introduction of the BCR-ABL TKIs in CML therapy has been a major advance in leukemia treatment. However, TKIs are ineffective in patients who undergo blastic transformation, and unable to eradicate CML at the stem cell level. Moreover, often clinical drug resistance can develop through the acquisition of BCR-ABL gene mutations [6], [7]. Given the promising *in vitro* results recently obtained with newly-shaped small molecules [12], in this study a systems biology approach was applied to assess global protein expression changes and targeted protein pathways upon treatment of K562 cells with the compounds **IND_S1, MEL_T1, IND_S7** and **MEL_S3**.

By 2-DE-based proteomic analysis, we identified several proteins that were differentially expressed in leukemia K562 cells incubated in the presence or absence of these new molecules. In particular, functional classification of the identified proteins revealed that they were mainly related to cellular metabolism, chaperone activity, cytoskeleton organization and RNA biogenesis. Interestingly, most of the proteins belonging to the first category were located in mitochondria and strongly down-regulated in **IND_S7**- and **MEL_S3**-treated K562 cells, suggesting an overall bioenergetics deficit that may contribute to counteract the well-known Warburg effect [24], triggering growth arrest and potentially apoptosis [25]. These data are in accordance with a previous study showing that all the four small molecules induced cell cycle arrest in K562 cells, whereas only **IND_S7** and **MEL_S3** exhibited pro-apoptotic ability [12]. Further, in all treatment groups of our study we found increased levels of two subunits of ATP synthase, whose activation may be a prerequisite for the energy-reliant apoptotic process to proceed [26]. On the other hand, we identified reduced levels of phosphoglycerate kinase 1 (after treatment with all the compounds), voltage-dependent anion-selective protein channel 2 (in K562 cells exposed to **IND_S1**, **IND_S7** and **MEL_S3**) and glutamate dehydrogenase 1 (in **MEL_S3**-treated K562 cells), which have been proposed to act as suppressors of apoptotic phenotypes [27], [28], [29].

In addition to the well documented chaperoning properties, a role as regulators of apoptosis has recently been attributed to heat shock proteins (HSP) [30]. Among the four major families of HSPs (HSP90, HSP70, HSP60 and the small HSPs, such as HSP27 and HSP10), proteins with anti-apoptotic (HSP27 and HSP70) and pro-apoptotic (HSP10 and HSP60) functions have been distinguished [31]. A large number of malignancies have been linked to the over-expression of HSPs, making them attractive targets for treatment strategies [32]. In particular, high endogenous levels of both constitutive and inducible HSP70 have been found in CML cells and associated with resistance to apoptosis by the chemotherapeutic drugs imatinib mesylate and nilotinib [33]. Intriguingly, all the compounds evaluated in our study induced a reduction of HSP70 expression at the transcript and protein level to a similar extent. This down-regulation was not associated to under-expression of the transcription factor HSF1, known to be the main regulator of the short-term induction of HSP [34], or rather, the mRNA abundances of HSF1 were found to be significantly up-regulated in **MEL_T1**-, **IND_S7**- and **MEL_S3**-treated K562 cells.

Both **IND_S7** and **MEL_S3** compounds were also able to reduce the levels of the alternative splicing regulator hnRNP L in K562 cells. Although aberrant expression and activity of certain hnRNPs have been already described [35], [36], suggesting that they may act as oncogenic factors [37], [38], the role of hnRNP L in CML is still largely unexplored. However, our findings are in agreement with a recent study that showed a significant decrease of hnRNP L expression in imatinib-resistant BCR-ABL-positive human cells after treatment with imatinib plus valproic acid [39]. Interestingly, Buchi *et al.* (2011) also reported profound post-translational modifications following combined treatment, with a significant induction of acetylation of hnRNP L.

To probe protein-protein interaction networks, which could predict the signaling pathways activated/deactivated under K562 cell treatment, and also to identify transcriptional factors and other low-abundance proteins classically not detectable in 2-DE gels, proteomic data were explored using MetaCore pathway analytical tools. Network analysis revealed a high number of interactions between differentially expressed proteins and various signaling factors. In particular, the top 3 transcriptional regulation networks showed SP1, c-Myc and HNF4-alpha as central hubs, suggesting that they might play a role in the pathophysiology of BCR-ABL-positive leukemias and/or represent therapeutic targets. Through qPCR validation of the resulting networks, the most interesting data on gene expression were obtained for **MEL_S3**, which was found to strongly induce mRNA levels of EGR1 and HNF4-alpha.

Given the involvement of EGR1 in differentiation and due to the relevance of differentiation as a possible therapeutic alternative in blastic crisis of CML, we were prompted to investigate the ability of the four synthetic small molecules to induce differentiation in K562 cells. Noteworthy, K562 cells treated with **MEL_S3** showed a marked expression of glycoprotein IIb/IIIa (CD41), an important cell marker in megakaryocytic differentiation. Several studies have demonstrated the involvement of EGR1 in regulating differentiation along a megakaryocyte lineage. For instance, both EGR1 and megakaryocytic differentiation have been shown to be rapidly induced in K562 and other leukemic cells treated with phorbol 12-myristate 13-acetate, with up-regulation of the megakaryocyte-specific gene CD41 and down-regulation of erythroid markers, such as glycophorin [19], [20], [21]. Analogously, in our study we observed a decreased expression of CD235a after K562 cell exposure to **MEL_S3**. The rate of megakaryocytic differentiation induced by the four synthetic small molecules was correlated to their ability to modulate EGR1 gene expression. In fact, **IND_S1** treatment, which resulted in a weaker increase of EGR1 transcript levels, determined a rate of megakaryocyte differentiation lower than that observed for **MEL_S3**. **MEL_T1**- and **IND_S7**-treated K562 cells, which showed a down-regulation of EGR1 mRNA abundances, were unable to differentiate along the megakaryocyte lineage.

K562 cells treated with **MEL-S3** also showed an increased expression of glycoprotein Ib (CD42), a component of the GPIb-V-IX complex on platelets, together with morphological characteristics of megakaryoblasts and megakaryocytes. Moreover, **MEL_S3** was able to induce megakaryocytic differentiation in HEL cells that, like to K562, are endowed with erythroid and megakaryocytic properties.

Finally, for the first time, our study demonstrated the presence of HNF4-alpha mRNA in K562 cells and a marked over-expression after exposure to **MEL_S3**. According to a model for its transcriptional transactivation ability, HNF4-alpha could activate p21 expression, mediating cell cycle arrest and apoptotic response, primarily by competing with c-Myc for interaction with promoter-bound SP1 [40]. On the other hand, c-Myc may not only repress p21 transcription by inhibiting the transcriptional activity and/or sequestering HNF4-alpha away from the promoter, but also indirectly activate p53 expression, leading to a p53-dependent induction of p21 [40], [41], [42]. These opposite effects reflect the dual role of c-Myc, whose over-activation is known to bring about continued cell-cycle progression and cellular immortalization, as well as to block differentiation and induce apoptosis [43]. In some systems, depending on the cell context and other activated molecular pathways, p21 and c-Myc have also been shown to synergize for driving post-mitotic megakaryocytic differentiation and polyploidy [44]. Interestingly, the highest mRNA abundances of c-Myc were observed in K562 cells exposed to **MEL_S3**, which could support the ability of this molecule to induce differentiation along the megakaryocyte lineage.

In conclusion, through a combination of proteomics and bioinformatics techniques, we were able to discover the pro-differentiation properties of a synthetic small molecule. The megakaryocyte differentiation induced by **MEL_S3** was correlated to its ability to up-regulate the EGR1 and HNF4-alpha transcript levels, the latter reported here for the first time to be present in K562 cells. Further studies are warranted to confirm **MEL_S3** contribution to CML management as well as to define its potential application in other malignancies characterized by similar pathway alterations.

## Materials and Methods

### Cell Culture

Continuous neoplastic K562 and HEL cells purchased from ATCC were kindly gifted by the Department of Biomedicines, University of Catania.

Cells were grown in RPMI 1640 (Gibco Grand Island, NY) containing 10% FCS (Gibco), 100 U/ml penicillin (Gibco), 100 µg/ml streptomycin (Gibco), and 2 mM L-glutamine (Sigma-Aldrich, St. Louis, MO) in a 5% CO2 atmosphere at 37°C.

### Sample Preparation

Each compound was dissolved in dimethyl sulfoxide (DMSO) in a stock solution at a concentration of 20 mM, stored at –20°C and protected from light. In each experiment DMSO never exceeded 0.2% and this percentage did not interfere with cell growth.

### Flow Cytometry

Flow cytometry was carried out to examine for the presence of erythroid and megakaryocyte cell surface markers on treated K562 and HEL cells. All monoclonal antibodies (mAbs) used were mouse IgG1 isotypes obtained from Becton Dickinson (San Jose, CA). The CD235a fluorescein isothiocyanate (FITC)-conjugated mAb was specific for glycophorin A, a major transmembrane sialoglycoprotein expressed on red blood cells and erythroid precursors. The CD41 mAb conjugated to phycoerythrin (PE) was specific for the GPIIb component of the GPIIb/IIIa complex present on platelets and early promegakaryoblasts [45], and CD42 FITC-conjugated mAb was specific for glycoprotein Ib, a component of the GPIb-V-IX complex on platelets. Each mAb was added in turn to approximately 5×10^5^ cells in 150 µl of RPMI/fetal bovine serum, lightly vortexed, and then incubated on ice for 45 min. After dilution with 2 ml of cold buffered RPMI/fetal bovine serum, cells were pelletted, resuspended in 400 µl of PBS and examined by a FACScan flow cytometer (Becton Dickinson).

### Morphological Evaluation of the Cells

Cells were spotted on cytospin slides by using a cytocentrifuge (Cytospin Heraeus Minifuge, Thermo Scientific, Waltham, MA), stained with May-Grunwald Giemsa and observed by using a Nikon Eclipse E200 microscope.

### Protein Extraction, Two-dimensional Gel Electrophoresis (2-DE) and Image Analysis

K562 cells incubated for 24 h in the presence or absence of 30 µM of each compound were rinsed in ice-cold washing buffer (3 mM KCl, 1.5 mM KH_2_PO_4_, 68 mM NaCl, 9 mM NaH_2_PO_4_) and lysed in lysis solution [0.11 M DTT, 0.11 M CHAPS, 8 M urea, 2 M thiourea, 35 mM Tris, supplemented with complete protease inhibitor cocktail (Roche, Mannheim, Germany)] for 15 min with constant vortexing. Whole-cell lysates were cleared by centrifugation at 9500 rpm for 1 h at 15°C, and the supernatants were reduced with tributylphosphine (Sigma-Aldrich) to a final concentration of 5 mM for 1 h, and then alkylated by 15 mM iodoacetamide (Sigma-Aldrich) for 1.5 h. Protein extracts were acetone precipitated and solubilized in IEF solution (7 M urea, 2 M thiourea, 4% CHAPS and 0.005% β-mercaptoethanol). Total protein concentration of the cell extracts was determined using the Bradford protein assay (Bio-Rad, Hercules, CA).

First-dimension isoelectric focusing was performed with IPGphor apparatus (GE Healthcare, Milan, Italy) as previously described [46]. Briefly, aliquots of samples containing equal quantities of protein (200 µg) were diluted to 250 µl with rehydration buffer [8 M urea, 2% CHAPS, 10 mM DTT, 1% (v/v) Pharmalyte (pH 3–10; GE Healthcare) and trace bromophenol blue] and applied onto 13-cm IPG strips (pH 3–10; GE Healthcare) for 12-h rehydration at 50 V. Running conditions were as follows: 1 h at 500 V, 1 h at 1000 V, 30-min ramp up to 8000 V, and 2 h at 8000 V for a total of 19 kVh. IPG strips were then reduced and alkylated [47] prior to loading onto 15% acrylamide gels. SDS-PAGE was carried out at 30 mA/gel using Protean II xi Cell (Bio-Rad). 2-DE gels were visualized with a MS-compatible silver-staining procedure [48] and scanned with a GS-800 imaging densitometer (Bio-Rad). Two biological replicates, each consisting of technical duplicates, were run.

Protein maps were analyzed by PDQuest v 8.0.1 software (Bio-Rad). Authenticity and outline of each spot were validated by eye and edited manually when necessary. Normalized spot volumes based on total quantity in valid spots were calculated for each 2-DE gel and used for statistical calculations of protein abundance.

### In-gel Digestion and MS

Spots of interest were excised and in-gel digested as previously described [49]. Crude digests were desalted and concentrated using a Poros Oligo R2 [PerSeptive Biosystems (Framingham, MA)] - GELoader microcolumn (Eppendorf, Hamburg, Germany), as reported by Gobom *et al*. [50]. The extracted peptides were dissolved in 0.1% trifluoroacetic acid (TFA) and 1/3 of the volume was applied to the R2 microcolumn, previously equilibrated with 10 µl of 0.1% TFA. The column was washed with 20 µl of 0.1% TFA, and retained peptides were eluted using 1 µl of *α*-cyano-4-hydroxycinnamic acid matrix solution (10 mg/ml) in 50% ACN with 0.1% TFA directly onto the MALDI plate and dried under ambient conditions.

All mass spectra were generated on a MALDI-TOF mass spectrometer Voyager DE PRO (Applied Biosystems), operating in positive ion reflectron (20 kV accelerating voltage) within the mass range 600–4000 Da. External mass calibration was performed with the signal of standard peptides (Sigma-Aldrich), achieving an accuracy in the measurement of peptide mass better than 60 ppm.

Peak lists were processed with GP-MAW software (http://welcome.to/gpmaw) and internally calibrated using keratin contaminant peptides and trypsin autolysis peptides. Re-calibrated peak lists were then submitted to the Mascot search engine (Matrix Science Ltd., London, UK) using the following criteria: database, NCBI (January 2012) or Swiss-Prot (release 2011_12); taxonomy, *Homo sapiens*; enzyme, Trypsin; fixed modifications, carbamidomethyl; variable modifications, oxidation on methionine and the *N*-terminal loss of ammonia at Gln; mass values, monoisotopic; parent tolerance, 0.2 Da; number of maximum missed cleavages, 1.

Proteins were considered as identified only when they had a significant Mowse Score and MW/pI similar to the experimental values found from the 2-DE gels. The candidates were further inspected for number and mass accuracy of matching peptides, sequence coverage and distribution of matching peptides in the obtained sequences.

### Bioinformatic Analysis of the Proteomic Data

The structure of raw proteomic data is known to be very disturbing for multivariate statistics and clustering algorithms, especially for the high rate of missing spot values [51]. To deal with this problem, only “reliable” protein spots were considered [i.e. given n experimental replications per condition, a protein spot was kept if at least (n−1) volume values were available for all samples] and missing values were imputed using the *k*-nearest neighbor (KNN) method (Matlab v 7.12.0, MathWorks, Torino, Italy).

To define differential spots, the dataset was subjected to uni- and multi-variate statistical analysis. Pairwise comparisons were performed using the classical statistical tests, Student’s t-test and the Mann-Whitney rank sum test (SigmaStat v 3.5 software, Systat Software, Point Richmond, CA). A *P* value<0.05 was considered as statistically significant. Multivariate data analysis methods were used for selecting significant spots and classifying the different group samples. Principal Component Analysis (PCA) was carried out using Canoco for Windows v 4.5 [52]. All variables were centered and weighted by (standard deviation)^−1^. Self-Organizing Map (SOM) clustering was performed by using the Neural Network toolbox for Matlab (MathWorks). The SOM algorithm is another way to reduce the dimensionality of the data, grouping those spots that change in the same way into an arbitrarily selected number of expression patterns. For SOM training, hexangular map lattice with unconnected edges and batch training mode were used as default parameters. A map size of 10×10 was chosen automatically by SOM based on the dimensions of the input data. The training length was set to 200 epochs (iterations).

Significant differences were analyzed through the two-way hierarchical clustering methodology using PermutMatrix v 1.9.3 software (http://www.lirmm.fr/~caraux/PermutMatrix/) [53]. Samples were clustered employing Ward’s minimum variance method over a Pearson distance-based dissimilarity matrix.

Protein accession numbers identified by MS analysis and their corresponding fold changes were imported into the web-based integrative software MetaCore™ (v 6.8 build 30387; Thomson Reuters, St. Joseph, MI) for network analysis. Networks were ranked by a *P* value and interpreted in terms of Gene Ontology (GO). Major hubs were identified based on the connections and edges within the networks.

### Western Blot Analysis

Primary antibodies used were: rabbit polyclonal anti-HSP70 (Cell Signaling Technology, Danvers, MA), rabbit monoclonal anti-GAPDH (Cell Signaling Technology), rabbit polyclonal anti-hnRNP L (Sigma-Aldrich) and mouse monoclonal anti-Sti1 (Sigma-Aldrich). Protein extracts (20 µg) were separated by conventional SDS-PAGE and transferred onto PVDF membranes. After electroblotting, membranes were blocked in a solution of 5% nonfat dry milk in TBS-T (PBS-T for anti-Sti1) for 1 h at RT, followed by incubation with primary antibody (1∶1000) o/n at 4°C. Excess antibodies were removed by three washings with T/PBS-T. Incubation with the appropriate secondary antibody conjugated to horseradish peroxidase (GE Healthcare) was performed at 1∶10000 dilution for 1 h at RT. After three washings, immunoreactive proteins were visualized using the ECL Plus detection system (GE Healthcare) on PhosphorImager Storm 840 (GE Healthcare). Chemiluminescence was quantified using ImageJ v 1.46 software (http://rsbweb.nih.gov/ij/) and Student’s t-test was applied to determine significance (*P*<0.05). GAPDH was used as an internal loading control.

### RNA Isolation, cDNA Synthesis and Quantitative Real-time PCR (qPCR)

Total RNA (2 µg), extracted according to standard procedures, was used as a template for reverse transcription reactions to synthesize single-stranded cDNA, using an oligo (dT) primer and MMLV-RT reverse transcriptase (Promega, Milan, Italy), following the manufacturer’s instructions.

qPCR was carried out with a LightCycler system (Roche) using SYBR Green to monitor cDNA amplification. One µl of cDNA template, equivalent to 50 ng RNA starting material, was used in each reaction along with 4 µl of SYBR Green PCR master mix (Roche) and 10 pmol of the appropriate gene-specific primers in a total volume of 20 µl. The following thermal profile was used: 10 min at 95°C, 40 repeats of 15 s at 95°C, 25 s at different annealing temperatures, 72°C for different elongation times and an additional 5-s incubation step at 80°C (92°C for HSF1) for fluorescence acquisition. Detailed primer-specific conditions and oligonucleotide sequence information are given in Table S1. Two technical replicates were done for each combination of cDNA and primer pair. Standard curves of the same RNA sample were registered for each gene, by analyzing two-fold serial dilutions of the cDNA. The amplification efficiency (E) was calculated for each primer pair using the equation E = [10^(−1/slope)^−1]×100. Product detection and PCR specificity were checked post-amplification by examining the temperature-dependent melting curves. The PCR products were resolved by 2% agarose gel electrophoresis to confirm the expected size of the cDNA fragments.

### qPCR Data Processing

Eight candidate reference genes were included: GAPDH, beta-2 microglobulin (B2M), mitochondrial ribosomal protein L19 (MRPL19), hypoxanthine phosphoribosyltransferase 1 (HPRT1), glucuronidase beta (GUSB), TATA-box-binding protein (TBP), BCR, ABL. Care was taken not to include genes of the same functional pathway to avoid problems of co-regulation and false positive reference gene selection. Gene expression stability was evaluated with two Microsoft Excel-based applications, the NormFinder MS Excel Add-in (http://www.mdl.dk/publicationsnormfinder.htm) [54] and the geNorm VBA applet for MS Excel (http://medgen.ugent.be/~jvdesomp/genorm) [17], following instructions included with the software downloads. The former application independently estimates the inter- and intra-group variance and combines both results in a stability value for each investigated gene. Genes with the lowest stability value have the most stable expression and will be top ranked. The other model-based approach, geNorm, tests for overall stability using a pairwise comparison method and uses the average pairwise variations to calculate the gene expression stability measure M. The higher the value of M, the higher the expression variability of the corresponding reference gene. According to M values, geNorm determines the optimal number of reference genes and computes a normalization factor (NF), based on the geometric mean of the expression levels of the best-performing reference genes, to be applied for subsequent quantifications. The relative expression levels of target genes were then reported as relative quantity of each gene against NF. Differences in gene expression levels between control and treated K562 cells were analyzed by Student’s t-test. *P*<0.05 was considered statistically significant.

## Supporting Information

Figure S1 2-DE protein profiles of K562 cells untreated (A) and after 24-h exposure to **IND_S1** (B), **MEL_T1** (C), **IND_S7** (D) and **MEL_S3** (E). IEF performed on IPG strips (13 cm, 3–10 linear pH gradient) was followed by second dimension separation on a 15% polyacrylamide gel. 2-DE gels were silver-stained. Identified spots, showing a significant difference between treated and control K562 cells with a ratio above 2 (circles) or under 0.5 (squares), are numbered (Table 1). Diamonds indicate protein spots with opposite trends of expression among treated K562 cells.
(TIF)

Figure S2 **Relative gene expression of selected proteins analyzed by qPCR.** Transcription levels were quantified according to Vandesompele *et al.* [17] (see text for details). Each bar represents mean ± SD of the fold ratio between treated and control K562 cells, derived from at least 2 separate experiments. *, *P*<0.05.
(TIF)

Figure S3 **Functional analysis of differentially expressed proteins from K562 cells exposed to IND_S1 (orange), MEL_T1 (blue), IND_S7 (red) and MEL_S3 (green).** (A) Enrichment of GeneGo pathway maps. (B) Enrichment of GeneGo process networks.
(TIF)

Figure S4 **Expression of selected transcription factors from network analysis at the mRNA level.** Transcription levels were quantified by qPCR according to Vandesompele*et al.* [17] (see text for details). Each bar represents mean ± SD of the fold ratio between treated and control K562 cells, derived from at least 2 separate experiments. *, *P*<0.05.
(TIF)

Figure S5 Expression of glycoprotein IIb/IIIa (A) andglycophorin A (B) in K562 cells cultured with or without 15 µM of **MEL_S3** (panel a), **MEL_T1** (panel b), **IND_S1** (panel c) and **IND_S7** (panel d). The expression of glycoprotein IIb/IIIa andglycophorin A was evaluated after 72 h of treatment by flow cytometry after staining cells with the monoclonal antibodies CD41 and CD235a. Thick line: untreated cells; thin line: treated cells.
(TIF)

Figure S6 **Expression of glycoprotein IIb/IIIa and glycoprotein Ib in K562 and HEL erythroleukemia cell lines.** Cells were cultured without (control) or with 15 µM of **MEL_S3**; the expression of glycoprotein IIb/IIIa and glycoprotein Ib was evaluated after 72 h of treatment by flow cytometry after staining cells with the monoclonal antibodies CD41 and CD42 respectively. a) expression of CD41 on K562 cells; b) expression of CD42 on K562 cells; c) expression of CD41 on HEL cells; d) expression of CD42 on HEL cells. Thick line: untreated cells; thin line: treated cells.
(TIF)

Table S1 qPCR primer sequences and amplification settings.
(DOC)

Table S2 Candidate housekeeping genes ranked by geNorm and NormFinder software according to their expression stability values. The lower the value, the greater the stability.
(DOC)

Table S3 Statistics of MetaCore network analysis of proteomic data and significant functional protein subnetworks using “analyze network” algorithm.
(DOC)

Table S4 Statistics of MetaCore network analysis of proteomic data and significant functional protein subnetworks using “transcription regulation” algorithm.
(DOC)
