# Supplementary figures and images for: A Natural-Like Synthetic Small Molecule Impairs Bcr-Abl Signaling Cascades and Induces Megakaryocyte Differentiation in Erythroleukemia Cells
Source: PLoS One. 2013 Feb 27;8(2):e57650. doi: 10.1371/journal.pone.0057650 (PMC3584047; doi:10.1371/journal.pone.0057650)

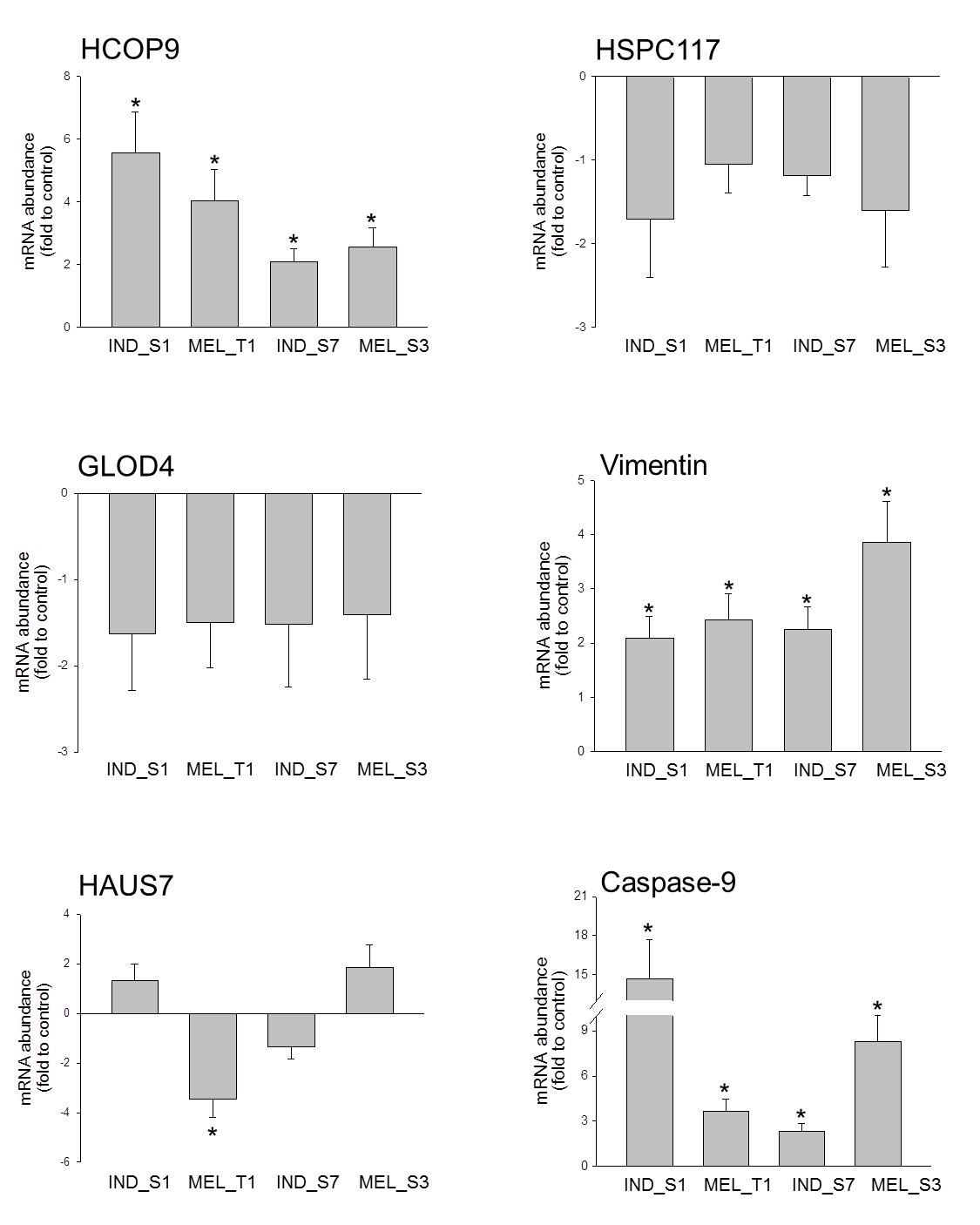

Supplement: Figure S2 — Relative gene expression of selected proteins analyzed by qPCR. Transcription levels were quantified according to Vandesompele et al. [17] (see text for details). Each bar represents mean ± SD of the fold ratio between treated and control K562 cells, derived from at least 2 separate experiments. *, P<0.05. (TIF) [file pone.0057650.s002.tif]

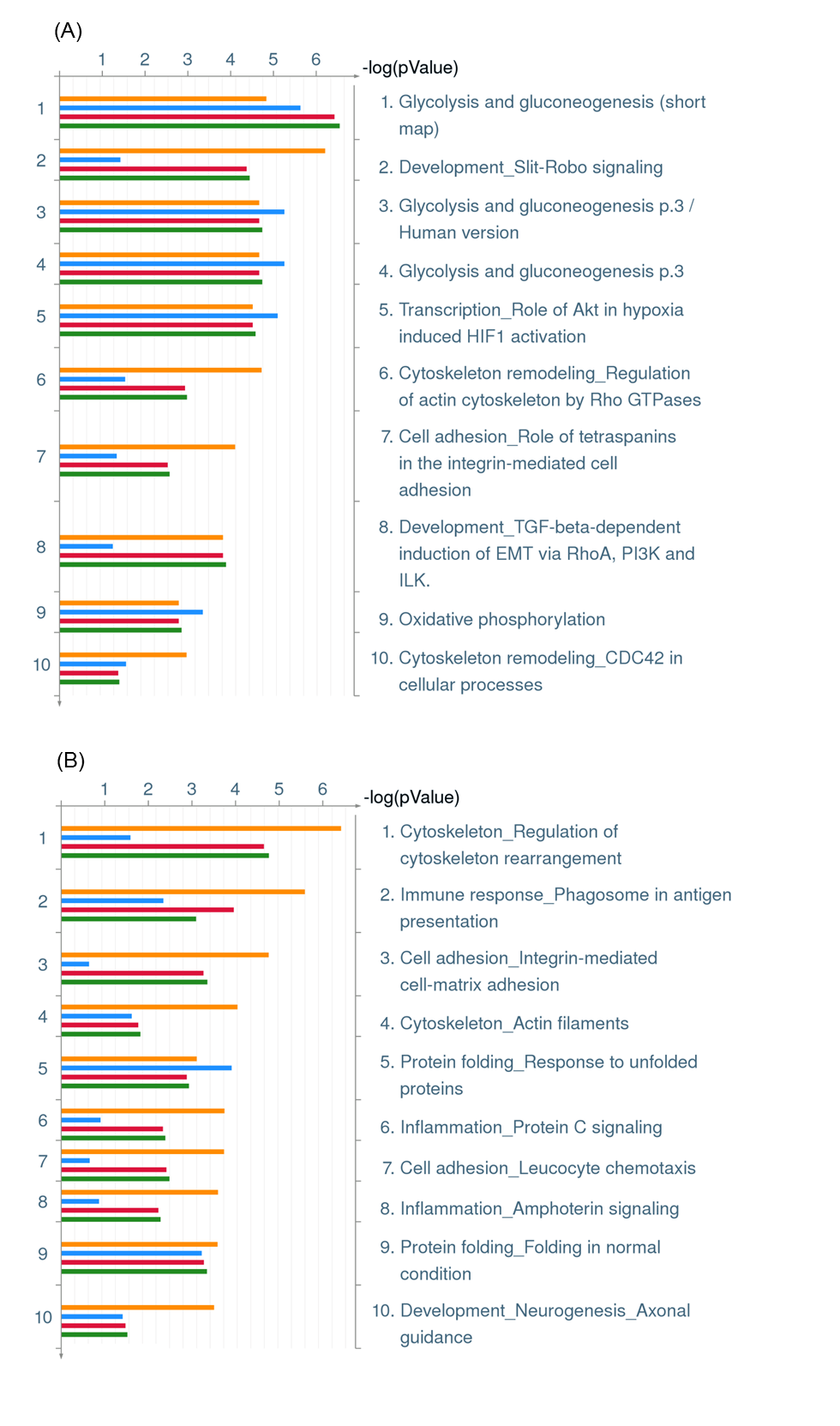

Supplement: Figure S3 — Functional analysis of differentially expressed proteins from K562 cells exposed to IND_S1 (orange), MEL_T1 (blue), IND_S7 (red) and MEL_S3 (green). (A) Enrichment of GeneGo pathway maps. (B) Enrichment of GeneGo process networks. (TIF) [file pone.0057650.s003.tif]

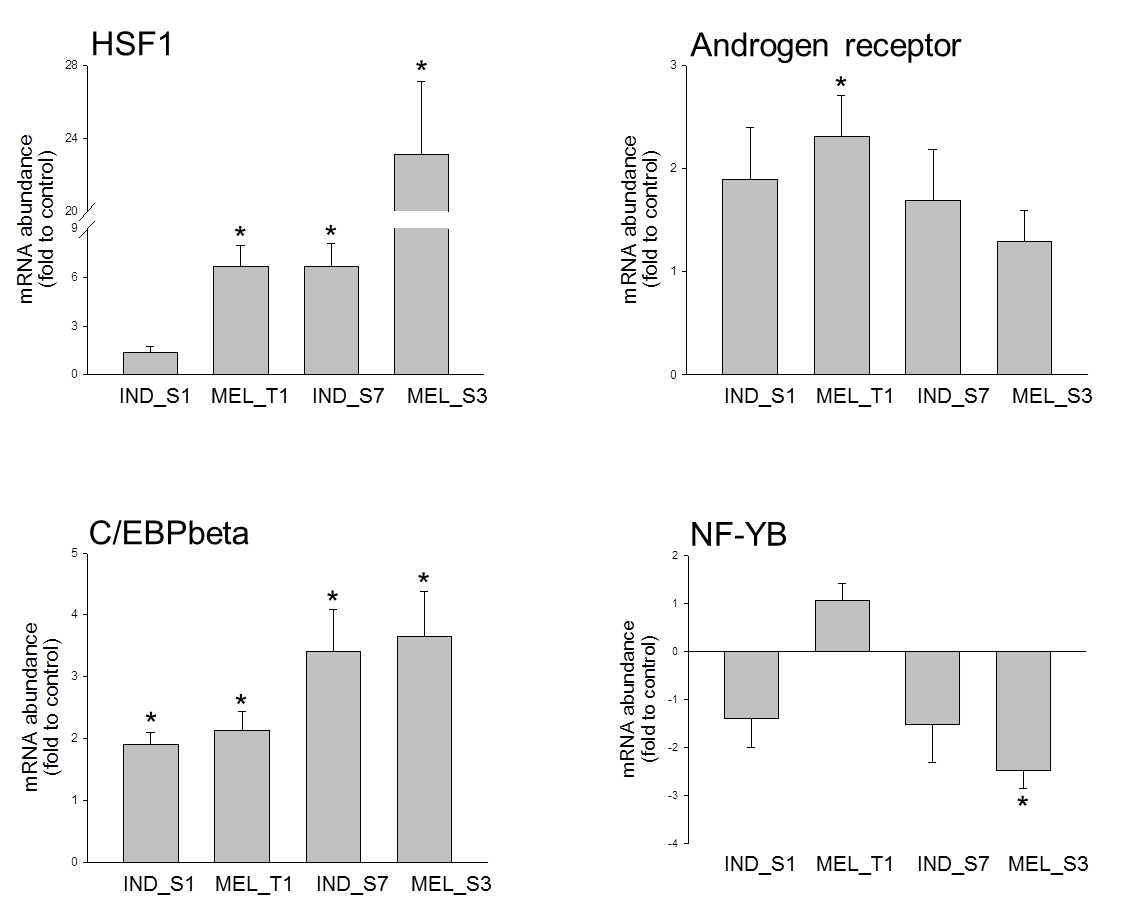

Supplement: Figure S4 — Expression of selected transcription factors from network analysis at the mRNA level. Transcription levels were quantified by qPCR according to Vandesompeleet al. [17] (see text for details). Each bar represents mean ± SD of the fold ratio between treated and control K562 cells, derived from at least 2 separate experiments. *, P<0.05. (TIF) [file pone.0057650.s004.tif]

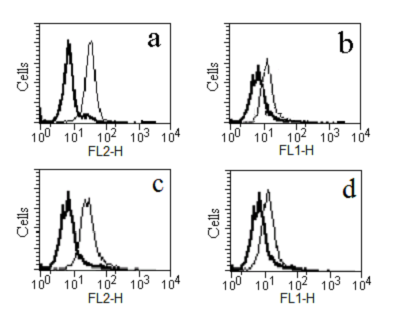

Supplement: Figure S6 — Expression of glycoprotein IIb/IIIa and glycoprotein Ib in K562 and HEL erythroleukemia cell lines. Cells were cultured without (control) or with 15 µM of MEL_S3; the expression of glycoprotein IIb/IIIa and glycoprotein Ib was evaluated after 72 h of treatment by flow cytometry after staining cells with the monoclonal antibodies CD41 and CD42 respectively. a) expression of CD41 on K562 cells; b) expression of CD42 on K562 cells; c) expression of CD41 on HEL cells; d) expression of CD42 on HEL cells. Thick line: untreated cells; thin line: treated cells. (TIF) [file pone.0057650.s006.tif]
