# Supplementary material for: A Natural-Like Synthetic Small Molecule Impairs Bcr-Abl Signaling Cascades and Induces Megakaryocyte Differentiation in Erythroleukemia Cells
Source: PLoS One. 2013 Feb 27;8(2):e57650. doi: 10.1371/journal.pone.0057650 (PMC3584047; doi:10.1371/journal.pone.0057650)
Supplement: Table S1 — qPCR primer sequences and amplification settings. (DOC) [file pone.0057650.s007.doc]

**Table S1.** qPCR primer sequences and amplification settings.

| Target gene | Sequence (5´-3´) | Fragment length (bp) | Annealing temp (°C) | Elongation time (s) | Efficiency (%) | Reference |
| --- | --- | --- | --- | --- | --- | --- |
| GAPDH | fwd:TGCACCACCAACTGCTTArev:GGATGCAGGGATGATGTTC | 177 | 60 | 20 | 80.3 |  |
| GUSB | fwd:GAAAATACGTGGTTGGAGAGCTCATTrev:CCGAGTGAAGATCCCCTTTTTA | 101 | 60 | 20 | 90.6 |  |
| B2M | fwd:CACCCCCACTGAAAAAGATGAGrev:CCTCCATGATGCTGCTTACATG | 106 | 60 | 20 | 100.6 |  |
| HPRT1 | fwd:ATGACCAGTCAACAGGGGACrev:TGCCTGACCAAGGAAAGCAA | 136 | 60 | 20 | 99.9 |  |
| MRPL19 | fwd:GGGATTTGCATTCAGAGATCAGGrev:CTCCTGGACCCGAGGATTATAA | 117 | 60 | 20 | 91.1 |  |
| TBP | fwd:TGCACAGGAGCCAAGAGTGAArev:CACATCACAGCTCCCCACCA | 132 | 60 | 20 | 90.0 |  |
| BCR | fwd:TCACCAAGAGAGAGAGGTCCAArev:CCTGCGATGGCGTTCAC | 202 | 60 | 20 | 79.0 |  |
| ABL | fwd:TCCTCGTCCTCCAGCTGTTATCrev:CCATTCCCCATTGTGATTATAGC | 246 | 60 | 20 | 88.5 |  |
| HSPA1 | fwd:GCTGCTGCTATTGCTTACGGCrev:GTACGGAGGCGTCTTACAGC | 263 | 60 | 20 | 95.3 |  |
| HNRNPL | fwd:TTCTGCTTATATGGCAATGTGGrev:GACTGACCAGGCATGATGG | 170 | 60 | 20 | 93.6 |  |
| STIP1 | fwd:CGGTATACTTTGAAAAGGGCGArev:CCGCTCTTGCTCCTTCAGGAT | 250 | 56 | 20 | 103.1 | This study |
| CASP9 | fwd:GCTCTTCCTTTGTTCATCTCCrev:CATCTGGCTCGGGGTTACTGC | 740 | 58 | 40 | 92.5 |  |
| HSF1 | fwd:ACCCATCATCTCCGACATCArev:CTACGCTGAGGCACTTTTCA | 340 | 60 | 25 | 96.6 | This study |
| HNF4A | fwd:CTGCTCGGAGCCACCAAGAGATCCATGrev:ATCATCTGCCAGGTGATGCTCTGCA | 371 | 60 | 25 | 94.4 | This study |
| AR | fwd:CCATTGAGCCAGGTGTAGTGTrev:CGAAGTAGAGCATCCTGGAGT | 250 | 62 | 20 | 107.3 | This study |
| MYC | fwd:GGGATCGCGCTGAGTATAAAArev:CCTCCTCGTCGCAGTAGAA | 530 | 60 | 30 | 100.7 | This study |
| CEBPB | fwd:AAACTCTCTGCTTCTCCCTCTGCrev:CTGACAGTTACACGTGGGTTGC | 130 | 62 | 20 | 84.5 |  |
| NFYA | fwd:GGAGGCCAGCTAATCACATCrev:GCCGAGACTCATGCAGGTAT | 711 | 60 | 40 | 102.6 |  |
| NFYB | fwd:AGGTGCCATCAAGAGAAACGrev:TGTTGTTGACCGTCTGTGGT | 250 | 60 | 20 | 90.8 | This study |
| NFYC | fwd:AGGTGCGCCAGTCTGTAACTrev:CCTTCTCCAACCTGCATTGT | 150 | 60 | 20 | 77.2 |  |
| EGR1 | fwd:CCCGTTCGGATCCTTTCCTrev:GTTTGGCTGGGGTAACTGGTCT | 310 | 58 | 20 | 75.0 | This study |
| SP1 | fwd:ATGGGGGCAATGGTAATGGTGGrev:TCAGAACTTGCTGGTTCTGTAAG | 410 | 60 | 25 | 90.6 |  |

**References**

1. Rho HW, Lee BC, Choi ES, Choi IJ, Lee YS, et al. (2010) Identification of valid reference genes for gene expression studies of human stomach cancer by reverse transcription-qPCR. BMC Cancer 10: 240.

2. Valceckiene V, Kontenyte R, Jakubauskas A, Griskevicius L (2010) Selection of reference genes for quantitative polymerase chain reaction studies in purified B cells from B cell chronic lymphocytic leukaemia patients. Br J Haematol 151: 232-238.

3. Shen Y, Li Y, Ye F, Wang F, Lu W, et al. (2010) Identification of suitable reference genes for measurement of gene expression in human cervical tissues. Anal Biochem 405: 224-229.

4. Branford S, Hughes TP, Rudzki Z (1999) Monitoring chronic myeloid leukaemia therapy by real-time quantitative PCR in blood is a reliable alternative to bone marrow cytogenetics. Br J Haematol 107: 587-599.

5. Du Q, Wang L, Zhu H, Zhang S, Xu L, et al. (2010) The role of heterogeneous nuclear ribonucleoprotein K in the progression of chronic myeloid leukemia. Med Oncol 27: 673-679.

6. Jones CD, Yeung C, Zehnder JL (2003) Comprehensive validation of a real-time quantitative bcr-abl assay for clinical laboratory use. Am J Clin Pathol 120: 42-48.

7. Yordy JS, Moussa O, Pei H, Chaussabel D, Li R, et al. (2005) SP100 inhibits ETS1 activity in primary endothelial cells. Oncogene 24: 916-931.

8. Zhang Z, Fan J, Becker KG, Graff RD, Lee GM, et al. (2006) Comparison of gene expression profile between human chondrons and chondrocytes: a cDNA microarray study. Osteoarthritis Cartilage 14: 449-459.

9. Hung LH, Heiner M, Hui J, Schreiner S, Benes V, et al. (2008) Diverse roles of hnRNP L in mammalian mRNA processing: a combined microarray and RNAi analysis. RNA 14: 284-296.

10. Goehe RW, Shultz JC, Murudkar C, Usanovic S, Lamour NF, et al. (2010) hnRNP L regulates the tumorigenic capacity of lung cancer xenografts in mice via caspase-9 pre-mRNA processing. J Clin Invest 120: 3923-3939.

11. Yang J, Bridges K, Chen KY, Liu AY (2008) Riluzole increases the amount of latent HSF1 for an amplified heat shock response and cytoprotection. PLoS One 3: e2864.

12. Liu ZJ, Wang G, Cai Y, Gu SZ, Zhang XB, et al. (2009) Androgen receptor CpG island methylation status in human leukemia cancer cells. Cancer Invest 27: 156-162.

13. Bergalet J, Fawal M, Lopez C, Desjobert C, Lamant L, et al. (2011) HuR-mediated control of C/EBPbeta mRNA stability and translation in ALK-positive anaplastic large cell lymphomas. Mol Cancer Res 9: 485-496.

14. Benatti P, Basile V, Merico D, Fantoni LI, Tagliafico E, et al. (2008) A balance between BF-Y and p53 governs the pro- and anti-apoptotic transcriptional response. Nucleic acid research 36: 1415-1428.

15. Cullen EM, Brazil JC, O'Connor CM (2010) Mature human neutrophils constitutively express the transcription factor EGR-1. Mol Immunol 47: 1701-1709.

16. Choi ES, Shim JH, Jung JY, Kim HJ, Choi KH, et al. (2011) Apoptotic effect of tolfenamic acid in androgen receptor-independent prostate cancer cell and xenograft tumor through specificity protein 1. Cancer Sci 102: 742-748.
