# Supplementary material for: A Natural-Like Synthetic Small Molecule Impairs Bcr-Abl Signaling Cascades and Induces Megakaryocyte Differentiation in Erythroleukemia Cells
Source: PLoS One. 2013 Feb 27;8(2):e57650. doi: 10.1371/journal.pone.0057650 (PMC3584047; doi:10.1371/journal.pone.0057650)
Supplement: Table S2 — Candidate housekeeping genes ranked by geNorm and NormFinder software according to their expression stability values. The lower the value, the greater the stability. (DOC) [file pone.0057650.s008.doc]

**Table S2.** Candidate housekeeping genes ranked by geNorm and NormFinder software according to their expression stability values. The lower the value, the greater the stability.

| Gene name | geNormM value | NormFinderstabilityvalue |
| --- | --- | --- |
| TBP | 0.613 | 0.167 |
| GAPDH | 0.652 | 0.184 |
| BCR | 0.665 | 0.205 |
| B2M | 0.670 | 0.240 |
| GUSB | 0.760 | 0.269 |
| MRPL19 | 0.845 | 0.346 |
| HPRT1 | 0.870 | 0.364 |
| ABL | 1.094 | 0.435 |
