# Supplementary material for: A Natural-Like Synthetic Small Molecule Impairs Bcr-Abl Signaling Cascades and Induces Megakaryocyte Differentiation in Erythroleukemia Cells
Source: PLoS One. 2013 Feb 27;8(2):e57650. doi: 10.1371/journal.pone.0057650 (PMC3584047; doi:10.1371/journal.pone.0057650)
Supplement: Table S3 — Statistics of MetaCore network analysis of proteomic data and significant functional protein subnetworks using “analyze network” algorithm. (DOC) [file pone.0057650.s009.doc]

**Table S3.** Statistics of MetaCore network analysis of proteomic data and significant functional protein subnetworks using “analyze network” algorithm.

| # | Network | GO processes | Seednodes | p-Value | zScore |
| --- | --- | --- | --- | --- | --- |
| 1 | HSPA1A, PDIA3, hnRNP L, KHSRP, ERp29 | regulation of apoptosis (55.1%; 4.693e-19), regulation of programmed cell death (55.1%; 5.823e-19), regulation of cell death (55.1%; 1.270e-18), response to stress (69.4%; 1.780e-17), response to organic substance (59.2%; 3.180e-16) | 9 | 4.160E-21 | 53.56 |
| 2 | VDAC 2, DHE3, ATP5B, PSMA1, ETFA | odontogenesis of dentine-containing tooth (17.5%; 1.127e-10), response to organic cyclic compound (27.5%; 6.253e-10), fungiform papilla formation (10.0%; 1.136e-09), fungiform papilla morphogenesis (10.0%; 1.136e-09), maintenance of chromatin silencing (10.0%; 1.891e-09) | 8 | 1.020E-18 | 49.63 |
| 3 | Vimentin, PDIA3, HSPA1A, ENO1, KHSRP | regulation of apoptosis (63.8%; 1.153e-23), regulation of programmed cell death (63.8%; 1.472e-23), regulation of cell death (63.8%; 3.563e-23), negative regulation of apoptosis (46.8%; 3.233e-20), negative regulation of programmed cell death (46.8%; 4.043e-20) | 6 | 2.680E-13 | 36.02 |
| 4 | HCOP9, hnRNP L, KHSRP, PRDX1, Sti1 | positive regulation of cellular biosynthetic process (70.2%; 2.322e-28), positive regulation of biosynthetic process (70.2%; 4.052e-28), positive regulation of RNA metabolic process (66.0%; 7.109e-28), positive regulation of nitrogen compound metabolic process (68.1%; 1.323e-27), positive regulation of transcription, DNA-dependent (63.8%; 6.071e-27) | 5 | 8.490E-11 | 29.68 |
| 5 | KHSRP, hnRNP L, HSP70, ERp29, Sti1 | response to organic substance (65.2%; 1.860e-18), response to stress (71.7%; 1.050e-17), regulation of immune system process (47.8%; 7.264e-17), regulation of apoptosis (52.2%; 2.343e-16), regulation of programmed cell death (52.2%; 2.834e-16) | 5 | 8.490E-11 | 29.68 |
| 6 | Ferrocytochrome C + O(,2) = Ferricytochrome C + H('+) + H(,2)O, Ferrocytochrome C intracellular, O(,2) intracellular, Cytochrome c oxidase, H('+) mitochondrion | respiratory electron transport chain (100.0%; 3.464e-05), electron transport chain (100.0%; 6.481e-05), cellular respiration (100.0%; 6.849e-05), energy derivation by oxidation of organic compounds (100.0%; 3.263e-04), generation of precursor metabolites and energy (100.0%; 5.942e-04) | 1 | 1.690E-03 | 24.32 |
| 7 | FUMH, Fumaric acid + H(,2)O = L-Malic acid, L-Malic acid cytoplasm, Fumaric acid intracellular | fumarate metabolic process (100.0%; 1.353e-04), malate metabolic process (100.0%; 3.608e-04), homeostasis of number of cells within a tissue (100.0%; 1.263e-03), tricarboxylic acid cycle (100.0%; 1.533e-03), acetyl-CoA catabolic process (100.0%; 1.578e-03) | 1 | 1.690E-03 | 24.32 |
| 8 | hnRNP L, HSP70, ENO1, PRDX1, p21 | muscle structure development (42.9%; 9.535e-22), regulation of protein modification process (53.1%; 4.461e-21), regulation of phosphorylation (51.0%; 5.704e-21), muscle tissue development (36.7%; 9.459e-21), regulation of phosphorus metabolic process (51.0%; 1.292e-20) | 4 | 1.870E-08 | 23.71 |
| 9 | PRDX2, PPCKM, RelA (p65 NF-kBsubunit), microRNA 21, GTP + Oxaloacetic acid = GDP + HCO(,3)('-) + Phosphoenolpyruvate | response to organic substance (65.9%; 4.476e-18), positive regulation of biological process (77.3%; 9.420e-18), response to wounding (52.3%; 2.397e-17), cGMP biosynthetic process (18.2%; 1.864e-16), response to stimulus (95.5%; 2.830e-16) | 3 | 2.750E-06 | 18.11 |
| 10 | HSP70, PRDX1, PTEN, GRP78, AUF1 | response to unfolded protein (17.8%; 9.326e-12), response to topologically incorrect protein (17.8%; 1.591e-11), response to organic substance (51.1%; 2.407e-11), regulation of apoptosis (42.2%; 3.708e-11), regulation of programmed cell death (42.2%; 4.288e-11) | 3 | 2.930E-06 | 17.92 |
| 11 | ACTB, HSP70, GC1QBP, Beta-catenin, JAK2 | response to organic substance (71.7%; 3.300e-22), regulation of apoptosis (60.9%; 2.384e-21), regulation of programmed cell death (60.9%; 2.990e-21), regulation of cell death (60.9%; 6.788e-21), regulation of phosphorylation (52.2%; 1.736e-20) | 3 | 2.930E-06 | 17.92 |
| 12 | ENO1, Sti1, SMAD3, HSC70, BMP2 | response to external stimulus (62.0%; 1.834e-23), positive regulation of macromolecule metabolic process (64.0%; 2.183e-22), positive regulation of cellular metabolic process (64.0%; 5.652e-22), cell differentiation (74.0%; 5.944e-22), developmental process (88.0%; 7.140e-22) | 3 | 3.110E-06 | 17.74 |
| 13 | Cofilin, non-muscle, GC1QBP, Androgenreceptor, C1q, Endoplasmin | response to chemical stimulus (74.5%; 2.471e-16), response to stress (68.1%; 3.770e-16), negative regulation of cellular process (66.0%; 6.828e-16), response to organic substance (59.6%; 8.139e-16), positive regulation of biological process (70.2%; 3.496e-15) | 2 | 3.690E-04 | 11.77 |
| 14 | PGK1, NF-kB, VISA, ATP + 3-Phospho-L-glyceric acid = ADP + 1,3-Diphospho-D-glycerate, 1,3-Diphospho-D-glycerate + ADP = ATP + 3-Phospho-D-glyceric acid | regulation of immune response (72.1%; 9.146e-37), regulation of immune system process (72.1%; 4.815e-31), antigen processing and presentation (41.9%; 2.234e-26), immune system process (74.4%; 1.616e-25), regulation of immune effector process (44.2%; 1.979e-24) | 1 | 2.440E-02 | 6.21 |

Gene Ontology (GO) explains the functional processes associated with built network.

zScore indicates association among the functional subnetworks of the differentially expressed proteins from 2-DE analysis.
