# Supplementary material for: A Natural-Like Synthetic Small Molecule Impairs Bcr-Abl Signaling Cascades and Induces Megakaryocyte Differentiation in Erythroleukemia Cells
Source: PLoS One. 2013 Feb 27;8(2):e57650. doi: 10.1371/journal.pone.0057650 (PMC3584047; doi:10.1371/journal.pone.0057650)
Supplement: Table S4 — Statistics of MetaCore network analysis of proteomic data and significant functional protein subnetworks using “transcription regulation” algorithm. (DOC) [file pone.0057650.s010.doc]

**Table S4.** Statistics of MetaCore network analysis of proteomic data and significant functional protein subnetworks using “transcription regulation” algorithm.

| # | Network | GO processes | Seednodes | p-Value | zScore |
| --- | --- | --- | --- | --- | --- |
| 1 | SP1 | negative regulation of inclusion body assembly (16.7%; 4.487e-09), regulation of inclusion body assembly (16.7%; 2.509e-08), catabolic process (61.1%; 4.986e-08), negative regulation of myeloid cell apoptosis (16.7%; 1.278e-07), negative regulation of vasoconstriction (16.7%; 1.626e-07) | 17 | 6.380E-57 | 169.00 |
| 2 | c-Myc | catabolicprocess (69.2%; 1.835e-07), gluconeogenesis (23.1%; 2.515e-06), glycolysis (23.1%; 4.058e-06), glucosemetabolicprocess (30.8%; 4.483e-06), hexosebiosyntheticprocess (23.1%; 4.768e-06) | 12 | 1.320E-39 | 140.36 |
| 3 | HNF4-alpha | dicarboxylic acid metabolic process (18.2%; 4.436e-04), negative regulation of protein import into nucleus, translocation (9.1%; 9.920e-04), fumarate metabolic process (9.1%; 1.488e-03), negative regulation of tyrosine phosphorylation of Stat5 protein (9.1%; 1.983e-03), regulation of microvillus assembly (9.1%; 1.983e-03) | 10 | 7.580E-33 | 127.15 |
| 4 | CREB1 | negative regulation of inclusion body assembly (22.2%; 1.464e-06), regulation of inclusion body assembly (22.2%; 4.096e-06), negative regulation of myeloid cell apoptosis (22.2%; 1.140e-05), negative regulation of vasoconstriction (22.2%; 1.329e-05), protein refolding (22.2%; 1.752e-05) | 8 | 3.560E-26 | 112.46 |
| 5 | AP-1 | interspecies interaction between organisms (44.4%; 4.427e-05), regulation of binding (33.3%; 8.279e-05), multi-organism process (55.6%; 1.364e-04), gluconeogenesis (22.2%; 1.568e-04), hexose biosynthetic process (22.2%; 2.393e-04) | 8 | 3.560E-26 | 112.46 |
| 6 | HSF1 | negative regulation of inclusion body assembly (42.9%; 1.926e-10), regulation of inclusion body assembly (42.9%; 1.078e-09), negative regulation of myeloid cell apoptosis (42.9%; 5.503e-09), negative regulation of vasoconstriction (42.9%; 7.003e-09), protein refolding (42.9%; 1.077e-08) | 6 | 1.360E-19 | 95.63 |
| 7 | GCR-alpha | regulation of apoptosis (57.1%; 6.825e-04), regulation of programmed cell death (57.1%; 7.048e-04), regulation of cell death (57.1%; 7.919e-04), protein import into nucleus (28.6%; 8.027e-04), nuclear import (28.6%; 8.374e-04) | 6 | 1.360E-19 | 95.63 |
| 8 | GATA-1 | response to stress (85.7%; 7.362e-05), positive regulation of protein modification process (42.9%; 3.577e-04), interspecies interaction between organisms (42.9%; 5.064e-04), multi-organism process (57.1%; 6.029e-04), positive regulation of cellular protein metabolic process (42.9%; 6.238e-04) | 6 | 1.360E-19 | 95.63 |
| 9 | Androgenreceptor | oxidation-reduction process (57.1%; 2.543e-04), cell redox homeostasis (28.6%; 2.668e-04), activation of prostate induction by androgen receptor signaling pathway (14.3%; 3.157e-04), male somatic sex determination (14.3%; 3.157e-04), negative regulation of integrin biosynthetic process (14.3%; 3.157e-04) | 6 | 1.360E-19 | 95.63 |
| 10 | ERR1 | ADP biosynthetic process (33.3%; 3.353e-06), respiratory electron transport chain (50.0%; 3.979e-06), purine ribonucleoside diphosphate biosynthetic process (33.3%; 4.023e-06), purine nucleoside diphosphate biosynthetic process (33.3%; 4.023e-06), ribonucleoside diphosphate biosynthetic process (33.3%; 4.753e-06) | 5 | 2.450E-16 | 86.08 |
| 11 | AP-2A | anatomicalstructurearrangement (33.3%; 5.545e-06), gluconeogenesis (33.3%; 6.561e-05), hexosebiosyntheticprocess (33.3%; 1.002e-04), 'de novo'posttranslationalproteinfolding (33.3%; 1.420e-04), 'de novo'proteinfolding (33.3%; 1.590e-04) | 5 | 2.450E-16 | 86.08 |
| 12 | EGR1 | nucleobase-containing compound metabolic process (100.0%; 1.357e-04), cellular nitrogen compound metabolic process (100.0%; 2.410e-04), positive regulation of glomerular metanephric mesangial cell proliferation (16.7%; 2.706e-04), nitrogen compound metabolic process (100.0%; 2.782e-04), response to norepinephrine stimulus (16.7%; 5.411e-04) | 5 | 2.450E-16 | 86.08 |
| 13 | RelA (p65 NF-kBsubunit) | negative regulation of inclusion body assembly (40.0%; 4.067e-07), regulation of inclusion body assembly (40.0%; 1.138e-06), negative regulation of myeloid cell apoptosis (40.0%; 3.170e-06), negative regulation of vasoconstriction (40.0%; 3.698e-06), protein refolding (40.0%; 4.876e-06) | 4 | 4.130E-13 | 75.43 |
| 14 | C/EBPbeta | Bergmann glial cell differentiation (20.0%; 9.018e-04), oxaloacetate metabolic process (20.0%; 2.703e-03), multi-organism process (60.0%; 2.732e-03), lens fiber cell development (20.0%; 3.153e-03), regulation of interleukin-6 biosynthetic process (20.0%; 4.053e-03) | 4 | 4.130E-13 | 75.43 |
| 15 | NRF2 | cellularresponse to oxidative stress (60.0%; 2.044e-06), cellularresponse to superoxide (40.0%; 3.170e-06), removal of superoxideradicals (40.0%; 3.170e-06), cellularresponse to oxygen radical (40.0%; 3.170e-06), response to superoxide (40.0%; 4.876e-06) | 4 | 4.130E-13 | 75.43 |
| 16 | E2F1 | negative regulation of transcription involved in G1/S phase of mitotic cell cycle (20.0%; 6.764e-04), Bergmann glial cell differentiation (20.0%; 9.018e-04), protein deneddylation (20.0%; 2.478e-03), cullindeneddylation (20.0%; 2.478e-03), lens fiber cell development (20.0%; 3.153e-03) | 4 | 4.130E-13 | 75.43 |
| 17 | SRF | blood coagulation (80.0%; 3.318e-06), coagulation (80.0%; 3.318e-06), hemostasis (80.0%; 3.445e-06), regulation of body fluid levels (80.0%; 7.238e-06), wound healing (80.0%; 8.049e-06) | 4 | 4.130E-13 | 75.43 |
| 18 | USF1 | glucosemetabolicprocess (60.0%; 7.350e-06), hexosemetabolicprocess (60.0%; 1.443e-05), monosaccharidemetabolicprocess (60.0%; 2.702e-05), gluconeogenesis (40.0%; 4.380e-05), hexosebiosyntheticprocess (40.0%; 6.691e-05) | 4 | 4.130E-13 | 75.43 |
| 19 | HIF1A | gluconeogenesis (40.0%; 4.380e-05), glycolysis (40.0%; 6.012e-05), hexosebiosyntheticprocess (40.0%; 6.691e-05), glucosecatabolicprocess (40.0%; 9.212e-05), monosaccharidebiosyntheticprocess (40.0%; 1.122e-04) | 4 | 4.130E-13 | 75.43 |
| 20 | HNF6 | negative regulation of cellular process (100.0%; 4.072e-04), negative regulation of biological process (100.0%; 5.989e-04), negative regulation of inclusion body assembly (25.0%; 9.018e-04), positive regulation of biological process (100.0%; 9.837e-04), anti-apoptosis (50.0%; 1.207e-03) | 3 | 6.470E-10 | 63.25 |
| 21 | SP3 | Bergmann glial cell differentiation (25.0%; 7.215e-04), camera-type eye development (50.0%; 1.207e-03), eye development (50.0%; 1.612e-03), enucleate erythrocyte differentiation (25.0%; 1.623e-03), embryonic process involved in female pregnancy (25.0%; 2.343e-03) | 3 | 6.470E-10 | 63.25 |
| 22 | SMAD3 | lens fiber cell differentiation (50.0%; 7.922e-06), lens development in camera-type eye (50.0%; 6.387e-05), negative regulation of wound healing (25.0%; 3.608e-04), positive regulation of transforming growth factor beta3 production (25.0%; 3.608e-04), negative regulation of cell growth (50.0%; 4.065e-04) | 3 | 6.470E-10 | 63.25 |
| 23 | NFIA | positive regulation of protein ubiquitination (50.0%; 2.561e-04), synaptonemal complex disassembly (25.0%; 3.608e-04), regulation of protein folding in endoplasmic reticulum (25.0%; 3.608e-04), regulation of protein ubiquitination (50.0%; 4.705e-04), proteasomal ubiquitin-dependent protein catabolic process (50.0%; 4.752e-04) | 3 | 6.470E-10 | 63.25 |
| 24 | c-Fos | cellularresponse to reactiveoxygenspecies (50.0%; 8.671e-05), cellularresponse to oxidative stress (50.0%; 2.094e-04), response to reactiveoxygenspecies (50.0%; 4.564e-04), Bergmannglialcelldifferentiation (25.0%; 7.215e-04), multi-organismprocess (75.0%; 1.151e-03) | 3 | 6.470E-10 | 63.25 |
| 25 | NRF1 | generation of precursor metabolites and energy (75.0%; 5.673e-05), respiratory electron transport chain (50.0%; 2.062e-04), electron transport chain (50.0%; 3.847e-04), cellular respiration (50.0%; 4.065e-04), energy derivation by oxidation of organic compounds (50.0%; 1.911e-03) | 3 | 6.470E-10 | 63.25 |
| 26 | ETS1 | negative regulation of cell adhesion involved in substrate-bound cell migration (25.0%; 7.215e-04), PML body organization (25.0%; 9.018e-04), nuclear body organization (25.0%; 1.082e-03), multi-organism process (75.0%; 1.151e-03), regulation of extracellular matrix disassembly (25.0%; 1.262e-03) | 3 | 6.470E-10 | 63.25 |
| 27 | HSF2 | negative regulation of inclusion body assembly (50.0%; 2.440e-07), regulation of inclusion body assembly (50.0%; 6.832e-07), negative regulation of myeloid cell apoptosis (50.0%; 1.903e-06), negative regulation of vasoconstriction (50.0%; 2.220e-06), protein refolding (50.0%; 2.927e-06) | 3 | 6.470E-10 | 63.25 |
| 28 | Sry | Bergmann glial cell differentiation (25.0%; 7.215e-04), positive regulation of male gonad development (25.0%; 1.262e-03), regulation of male gonad development (25.0%; 1.623e-03), oxaloacetate metabolic process (25.0%; 2.163e-03), cellular response to superoxide (25.0%; 2.343e-03) | 3 | 6.470E-10 | 63.25 |
| 29 | p53 | ER overload response (50.0%; 2.561e-06), cellular response to glucose starvation (50.0%; 3.731e-06), mitochondrial membrane organization (50.0%; 2.862e-05), negative regulation of transforming growth factor beta receptor signaling pathway (50.0%; 3.103e-05), response to endoplasmic reticulum stress (50.0%; 3.482e-05) | 3 | 6.470E-10 | 63.25 |
| 30 | NF-Y | protein folding (100.0%; 1.935e-08), cellular protein metabolic process (100.0%; 3.678e-04), protein metabolic process (100.0%; 7.610e-04), negative regulation of inclusion body assembly (25.0%; 9.018e-04), regulation of inclusion body assembly (25.0%; 1.443e-03) | 3 | 6.470E-10 | 63.25 |

Gene Ontology (GO) explains the functional processes associated with built network.

zScore indicates association among the functional subnetworks of the differentially expressed proteins from 2-DE analysis.
